# Crop suitability analysis for the coastal region of India through fusion of remote sensing, geospatial analysis and multi-criteria decision making

**DOI:** 10.1038/s41598-025-90754-1

**Published:** 2025-03-13

**Authors:** Nishtha Sawant, Bappa Das, Gopal Mahajan, Sujeet Desai, Anurag Raizada, Parveen Kumar, Pooja Singh

**Affiliations:** 1 ICAR-Central Coastal Agricultural Research Institute, Old Goa, Goa 403402 India; 2 University of California Davis, 1 Shields Ave, Davis, CA 95616 USA

**Keywords:** AHP, Crop suitability analysis, RS, GIS, Coastal India, Rice, Coconut, Environmental impact, Environmental monitoring

## Abstract

Crop suitability analysis plays an important role in identifying and utilizing the areas suitable for better crop growth and higher yield without deteriorating the natural resources. The present study aimed to identify suitable areas for rice and coconut cultivation across the coastal region of India using the analytic hierarchy process (AHP) integrated with geographic information systems (GIS) and remote sensing. A total of nine parameters were selected for suitability analysis including elevation, slope, soil depth, drainage, texture, pH, soil organic carbon, rainfall, temperature and a land use land cover (LULC) constraint map. This study represents the first-ever application of an integrated approach combining AHP, GIS, and remote sensing for crop suitability analysis in entire coastal region of India. The weights for the parameters and subclasses were assigned using the AHP method based on experts’ opinions. Subsequently, all the thematic maps were overlaid using the weighted overlay analysis to generate a land suitability map. Separately, the LULC crop mask map was used to extract suitable areas for rice and coconut cultivation to create crop-specific suitability maps. The final suitability maps were classified into four different classes: highly suitable, moderately suitable, marginally suitable, and not suitable for crop production. The findings revealed that approximately 13.68% of the study area was highly suitable, with around 19.26% and 18.35% being moderately and marginally suitable, respectively, and 13.76% was not suitable for rice cultivation. Similarly, for coconut cultivation, approximately 11% were highly suitable, with 27.40% and 18.34% being moderately and marginally suitable. However, about 35% of the total study region was deemed permanently unsuitable for any type of cultivation. The suitability maps were validated using area under receiver operating characteristic curve (AUROC). The AUROC values for rice and coconut were found to be 0.764 and 0.740 indicating high accuracy. By strategically cultivating rice and coconut in highly and moderately suitable locations identified in the current study, and utilizing marginally suitable areas for other crops, it is possible to achieve financial viability in agricultural production by increasing crop yield without causing harm to natural resources.

## Introduction

The coastal region of India is characterized by a rich diversity of climate, topography, soils, crops, livestock, fisheries, etc. This region, located between the sea and the ocean, is the most vulnerable to climate change. The crop and livestock productivity in this region is poor compared to the inland areas despite abundant natural resources^1^. Rice and coconut are the major crops grown along the coast of India; however, rice and coconut productivity of coastal districts are low (2719 kg ha^−1^ and 8147 nuts ha^−1^, respectively) as compared to overall Indian productivity (4058 kg ha^−1^ and 8,966 nuts ha^−1^)^2,3^. This productivity gap can be minimized by identifying suitable areas for rice and coconut in the coastal region of India where the potential production of these crops can be achieved. One effective method for increasing production and productivity is to use crop suitability analysis to identify more suitable and newer areas. Crop suitability analysis is performed to find the region most suitable for growing crops efficiently under the existing environmental conditions such as soil, climate, water, and nutrient availability. It is particularly crucial in the context of climate change and its impact on coastal regions, which could significantly affect crop productivity.

Crop suitability analysis plays a vital role in agricultural development and planning. To cater for the increasing demands for food due to population growth, environmental pollution, and climate change, it is crucial to determine the suitability of crops. Crop production without a thorough land suitability assessment can lead to land degradation. The idea of sustainable agriculture entails growing high-quality crops that are both cost-effective and environmentally and socially acceptable. This means that natural resources are used in the best possible way to achieve effective agricultural output. To have an efficient agricultural production system, it is necessary to have a suitable land allocation with good planning and timely management of the agricultural areas. These allocation plans can be created by assessment of the land’s potential and suitability for specific crops which are affected by features such as elevation, slope, soil, land cover, and climatic conditions^4^. The interplay of all these variables determines whether a certain place is suitable for growing a specific crop. Therefore, it is crucial to periodically assess the suitability of the land to develop a productive crop production system.

Conventionally crop suitability analysis is performed through field sampling which is time-consuming and cost-intensive. With the availability of open-source data and software, remote sensing (RS) and geographic information systems (GIS) based methods can map the crop suitability over large areas in a cost-efficient manner. Analytical hierarchy process (AHP) is one of the multi-criteria decision-making (MCDM) methods which is used to find suitable sites based on different parameters using RS and GIS^5–9^. Datasets namely soil physical and chemical properties such as texture, depth, drainage, pH, organic carbon, electrical conductivity, cation exchange capacity (CEC), soil moisture, terrain characteristics like elevation, and slope, along with the climate data viz. rainfall, temperature and potential evapotranspiration (PET), and land use land cover (LULC) map have been previously considered to perform this analysis^5,8,10–14^. To create the suitability map of various crops, all these databases were combined using the multi-criteria evaluation (MCE) method in GIS environment. In addition to the AHP-based MCDM method, approaches like machine learning, fuzzy and, frequency ratio have also been used to identify suitable sites^15–21^. AHP has been used in this study because of its simplicity, flexibility and robustness in solving complex decision making problems, by breaking it into a hierarchical structure^22^. It can handle both qualitative and quantitative data, making it highly flexible. AHP ensures consistency in evaluations via a stringent mathematical methodology, resulting in reliable outcomes. Furthermore, AHP works well with limited data under resource-constrained environments, providing a pragmatic alternative to data-intensive techniques such as machine learning or multi-criteria optimization. AHP is particularly valuable for agricultural studies as it combines experiential knowledge with quantitative and qualitative data for more holistic decision-making. Additionally, it provides a transparent decision-making process, crucial for effectively communicating findings to stakeholders and policymakers as compared to black box machine learning or deep learning models^23^.

Although AHP integrated with GIS and RS methods has been widely utilized to map crop suitability in various regions globally, its application to a larger and more diverse area, such as the Indian Coastal Region (ICR), has not been thoroughly evaluated. This study aims to bridge that gap by identifying suitable land areas for rice and coconut production along the coast of India. By integrating multiple factors including terrain, soil, and climatic characteristics into a GIS environment using the AHP method, this research seeks to provide a comprehensive analysis of crop suitability in the ICR. This approach not only helps in understanding the spatial distribution of optimal crop-growing regions but also aids in strategic planning for agricultural practices in response to environmental changes and resource availability.

## Materials and methods

### Study area

**Fig. 1:**
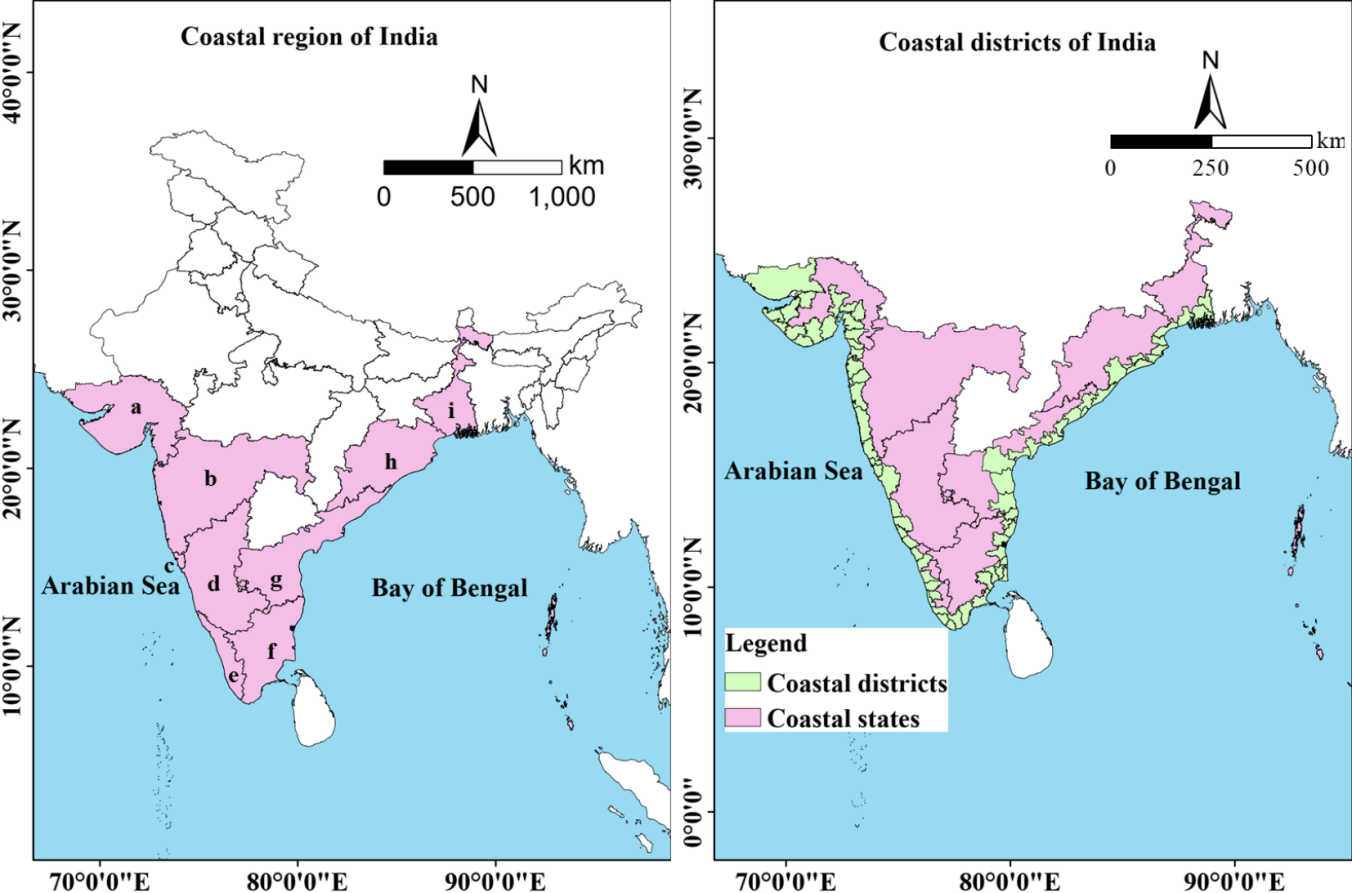
Location map highlighting coastal states and districts of India. (**a**) Gujarat, (**b**) Maharashtra, (**c**) Goa, (**d**) Karnataka, (**e**) Kerela, (**f**) Tamil Nadu, (**g**) Andhra Pradesh, (**h**) Odisha and (**i**) West Bengal (The maps were generated using ArcGIS software version 9.1 https://www.arcgis.com/index.html).

India has a very long coastline that extends for about 7516.6 km with the Bay of Bengal in the east, the Indian Ocean in the south, and the Arabian Sea in the west with a total area of 328,833.70 km^2^ (Fig. 1). The coastal districts account for a population of 188 million, which is 15.5% of the total national population (2011 Census). The mainland coastline of India consists of nine states namely Gujarat, Maharashtra, Goa, Karnataka, Kerala, Tamil Nadu, Andhra Pradesh, Odisha, and West Bengal, and two Union Territories which are Dadra and Nagar Haveli and Daman and Diu, and Puducherry. The majority of the population is dependent on agriculture for their livelihood in this region. The soils of the ICR are mostly fertile which is ideal for agriculture. Rice and coconut are the major crops of this region. The coastal region experiences a mean annual temperature (MAT) ranging from 23.76 to 28.91 °C, coupled with a mean annual rainfall range between 350.09 and 5043.19 mm. Enclosed by the coastline and hills on both sides, the coastal plains gradually ascend from the sea level toward the hills, with elevations ranging from  -5 to 2500.77 m.

### Data used

The primary datasets used in the study include topographical, climate, soil and LULC data (Table 1). These datasets were gathered from different sources with varying resolutions and coordinate systems. Nine parameters including elevation, slope, soil texture, soil drainage, soil depth, soil pH, soil organic carbon (SOC), rainfall and temperature were considered for the suitability analysis. The rationale behind selection of these parameters is that rice thrives in lowland areas with clayey, poorly drained soils favouring standing water, slightly acidic to neutral pH, and temperatures between 20 and 35 °C, requiring consistent rainfall^24^. Coconut, on the other hand, prefers sandy loam soils with good drainage, moderate slopes, and tropical conditions with temperatures around 27–30 °C and annual rainfall of 1500–2500 mm^25,26^. Both crops benefit from deeper soils for root development, higher SOC for fertility, and proper pH for nutrient availability, emphasizing the importance of these soil properties^27–30^. These parameters were classified based on the crop growth requirements. The weights were allocated to all the subclasses and main parameters based on AHP method. The suitability maps were obtained by overlaying all the thematic layers using weighted overlay analysis^31–33^.

**Table 1:** Covariates used in the study.

Dataset	Variables	Source of data	Spatial resolution/scale
SRTM DEM	Elevation, Slope	https://srtm.csi.cgiar.org/srtmdata/	90 m
Soil Data	Soil depth, soil drainage	Indian Council of Agricultural Research-National Bureau of Soil Survey & Land Use Planning (ICAR-NBSS&LUP), Nagpur, India	1:50,000
SoilGrids	Soil pH, SOC, Soil texture	https://soilgrids.org	250 m
WorldClim	Temperature (1990–2018)	https://www.worldclim.org	2.5 min
IMD	Rainfall (1901–2018)	https://www.imdpune.gov.in/lrfindex.php	0.25°
ESRI 2022 Land Cover	LULC	https://livingatlas.arcgis.com/landcover/	10 m

SRTM DEM, shuttle radar topography mission-digital elevation model; IMD, India Meteorological Department; ESRI, Environmental Systems Research Institute.

The filled Shuttle Radar Topography Mission (SRTM)-Digital Elevation Model (DEM) data was downloaded from the Consultative Group on International Agricultural Research-Consortium for Spatial Information (CGIAR-CSI)^34^. Mosaicking and masking of filled SRTM data was performed to get elevation data for the study area. Finally, the elevation raster was reprojected to Lambert Conformal Conic (LCC) projection system with a false easting and northing of 3,000,000, the central meridian of 79.5, latitude of origin of 16 and standard parallel 1 and 2 of 11.5 and 20.5, respectively with WGS 1984 datum at 250 m spatial resolution. Slope was calculated using the projected elevation data in percentage rise.

Sand, silt, clay, pH and soil organic carbon (SOC) for 0–5 cm and 5–15 cm soil depth with 250 m resolution were downloaded from SoilGrids using Google Earth Engine. The depth-weighted average of all the above parameters was taken to represent a 0–15 cm soil layer. The soil textural classes were calculated from sand, silt and clay contents following the USDA scheme in SAGA GIS software^35^. All of these parameters were also reprojected to LCC projection. The soil base maps at 1:50,000 scale were purchased from the ICAR-National Bureau of Soil Survey & Land Use Planning (ICAR-NBSS&LUP). The base maps were georeferenced and digitized to create vector files of soil drainage and depth. The soil drainage and depth vector files were subsequently rasterized at a spatial resolution of 250 m and projected to the LCC projection.

The mean monthly minimum and maximum temperature (°C) data at 2.5 min spatial resolution for the period 1990–2018 were downloaded from WorldClim^36 because of its relatively high spatial resolution^. The daily precipitation (mm) data available from the India Meteorological Department (IMD) from 1901 to 2018 at 0.25° spatial resolution were used in the study as it best represents the precipitation pattern of India^37^. The monthly minimum and maximum temperature data were converted to monthly climatological mean temperature by taking the mean of each month separately across all the years. Daily precipitation data was converted to cumulative monthly precipitation which was then averaged across years to get monthly climatological precipitation. To calculate mean annual precipitation, the sum of all 12 monthly rasters was computed. The mean temperatures for June, July, August, and September were averaged to calculate the rice growing season mean temperature, while the mean annual temperature was used for coconut suitability analysis. Further, the inverse distance weighted (IDW) method was applied to downscale the annual rainfall and mean temperature data to 250 m spatial resolution after assigning LCC projection. The optimum power of IDW was obtained using the “optimize” function in R software version 4.3.1^38^ which was 10.047 and 7.612 for temperature and precipitation, respectively.

LULC data for the year 2022 was downloaded from Environmental Systems Research Institute (ESRI) global land cover data at native resolution (10 m)^39^. The LULC data was then mosaicked, masked and reprojected to the LCC projection system at 250 m spatial resolution using nearest neighbor resampling. The LULC map was used to mask out the areas under waterbodies, built areas and forests from the final crop suitability map.

### Analytical hierarchy process (AHP)

The AHP approach was used to determine the relative significance of the criteria and sub-criteria. Allocation of accurate weights to parameters is one of the decisive aspects of AHP. It is mandatory to assign the relative weight for each criterion in the decision-making technique^40^. The two factors were compared to the suitability of the crop using a pairwise comparison matrix (PWCM) based on expert advice to determine the factor weights concurrently. The PWCM was implemented using a scale created by Saaty in 1980 with values ranging from 9 to 1/9 to determine the relative importance/weight of criteria and sub-criteria^40^. A score of 9 means that the row factor is more significant than the column factor. A value of 1/9, on the other hand, denotes that the row factor is less significant than the column element. When both the column and row factors are of equal importance, they get the same rating value of 1.

Since the matrix is symmetrical, any triangular half of the matrix needs to be filled and the remaining portions are just the reciprocals of filled values. When the same parameters are compared with one another, a value of unity is assigned diagonally. Furthermore, the individual parameter values in the matrix were divided by their corresponding sum of the columns to create the normalized comparison matrix. Later, the weights of individual parameters were generated based on the row total of the normalized comparison matrix divided by the total number of parameters^5,31,41,42^.

For AHP method, consistency should be maintained in PWCM. Hence, the Consistency Index (CI) was calculated for the matrix by applying Eq. (1).

1 $${\text{CI}} = \frac{{\left( {{\uplambda }_{{{\text{max}}}} { } - {\text{ n}}} \right)}}{{\left( {{\text{n }} - { }1} \right)}}$$

where ${\lambda }_{max}$ is the maximum eigenvalue, n is the number of criteria of the comparison matrix. The eigenvalues ($\lambda$) were calculated in Microsoft Excel using MMULT function. After calculating CI, the Consistency Ratio (CR) was determined with the Random consistency Index (RI), for a particular number of parameters (n) using Eq. (2) (Table 2).

2 $${\text{CR}} = \frac{{{\text{CI}}}}{{{\text{RI}}}}$$

**Table 2:** Random consistency index (RI) for N = 10 (*Source*: Saaty^40^).

N	1	2	3	4	5	6	7	8	9	10
RI	0	0	0.58	0.9	1.12	1.24	1.32	1.41	1.46	1.49

If the assessment of PWCM is accurate, then the CR value will be less than 0.1. The pairwise comparison matrix for rice and coconut suitability using AHP is presented in Table 3 and 4.

### Site suitability based on weighted overlay analysis

**Fig. 2:**
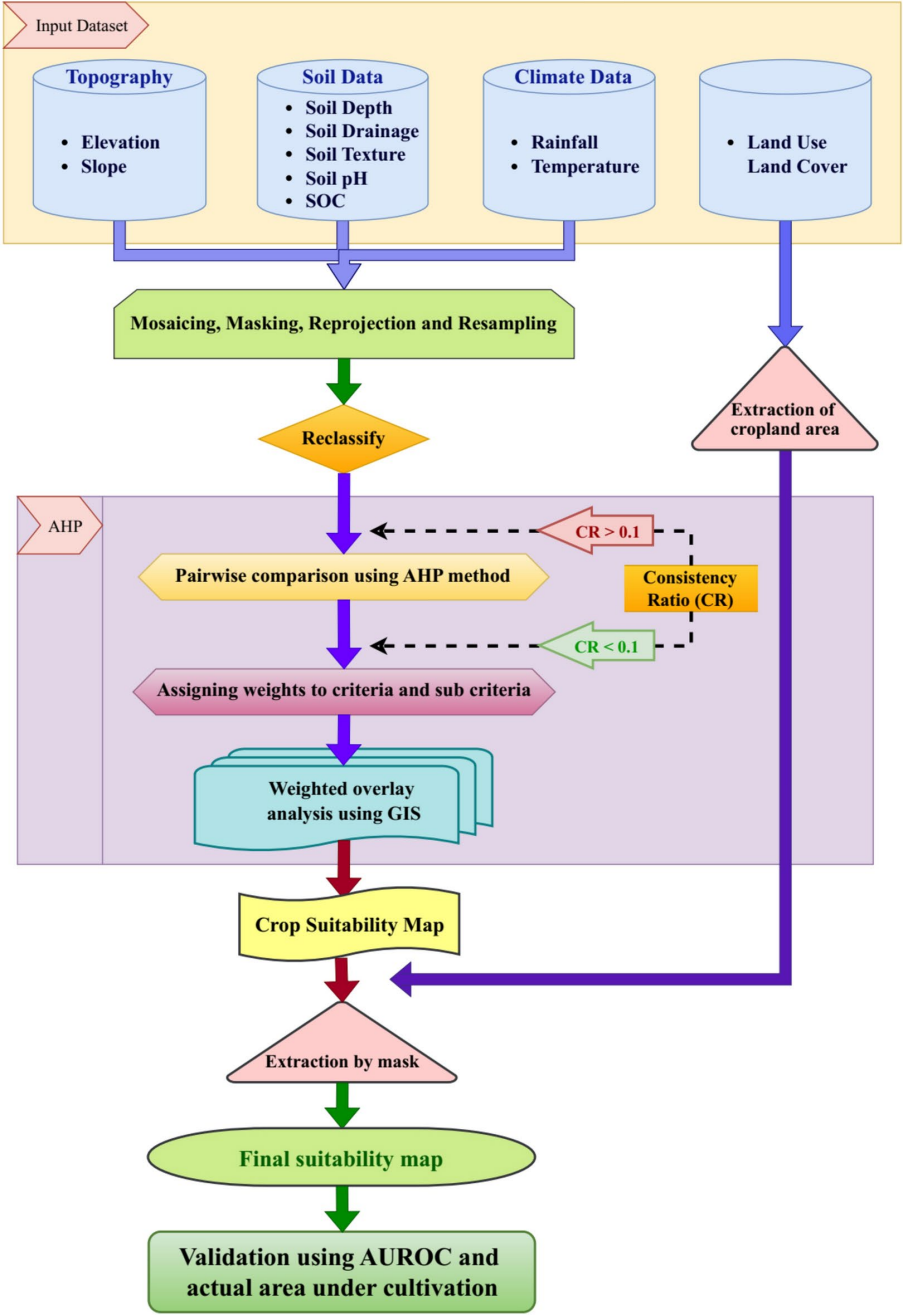
Overall methodology used in the current study.

All the nine parameters along with LULC were resampled to 250 m spatial resolution with 9333 rows and 7486 columns and were projected to the LCC projection system. The parameters were reclassified into subclasses to create the thematic layers separately for rice and coconut by considering the suitable conditions for their growth and development. Once all the thematic maps and assessments of weightage for all parameters and their subclasses were completed, weighted overlay analysis was performed to identify the best suitable sites for each crop using ArcGIS software. The suitability map was reclassified into highly suitable (S1), moderately suitable (S2), marginally suitable (S3) and not suitable (N) using equal interval classification and the area under each class was computed. The overall methodology used for the current study is presented in Fig. 2.

### Validation

For validation of suitability maps, the occurrence data of rice and coconut were downloaded from Global Biodiversity Information Facility (GBIF, https://www.gbif.org/). The occurrence records with geographic coordinates falling within the study area were selected after removal of duplicate records. After cleaning, we obtained 180 and 1481 presence records for rice and coconut, respectively. In addition, equal number of random points designated as absence for rice and coconut were created using “randomPoints” function from “dismo” R package^43^ leaving highly suitable areas. Then the suitability values for rice and coconut were extracted from crop suitability maps using the presence-absence coordinates. This dataset was used to compute area under receiver operating characteristic curve (AUROC). The AUROC values above 0.9 indicate very high accuracy, values between 0.7 and 0.9 signify high accuracy, while values below 0.7 denote low accuracy^44^.

**Table 3:** Pairwise comparison matrix of rice suitability using AHP.

	Rainfall	Temperature	Soil drainage	Soil depth	Soil texture	Slope	Elevation	SOC	Soil pH	Weights
Rainfall	1.00	8.00	1.00	5.00	3.00	6.00	6.00	7.00	9.00	0.29
Temperature	0.13	1.00	0.13	0.20	0.17	0.33	0.33	0.50	2.00	0.03
Soil drainage	1.00	8.00	1.00	3.00	2.00	5.00	5.00	7.00	9.00	0.25
Soil depth	0.20	5.00	0.33	1.00	0.50	3.00	3.00	4.00	7.00	0.11
Soil texture	0.33	6.00	0.50	2.00	1.00	4.00	4.00	5.00	7.00	0.16
Slope	0.17	3.00	0.20	0.33	0.25	1.00	1.00	2.00	5.00	0.06
Elevation	0.17	3.00	0.20	0.33	0.25	1.00	1.00	2.00	5.00	0.06
SOC	0.14	2.00	0.14	0.25	0.20	0.50	0.50	1.00	3.00	0.04
Soil pH	0.11	0.50	0.11	0.14	0.14	0.20	0.20	0.33	1.00	0.02

Consistency index (CI) = $({\lambda }_{max}-n )/(n-1)$  = 0.1140.

Number of parameters (n) = 9.

Maximum Eigenvalue $${(\lambda }_{\text{max}})= 9.9122$$.

Random index (RI) = 1.46.

Consistency ratio (CR) = CI/RI = 0.0781.

**Table 4:** Pairwise comparison matrix of coconut suitability using AHP.

	Elevation	Slope	Soil depth	Soil texture	Soil drainage	Rainfall	Temperature	Soil pH	SOC	Weight
Elevation	1.00	0.50	0.20	0.33	0.25	0.14	0.17	2.00	3.00	0.04
Slope	2.00	1.00	0.25	0.50	0.33	0.17	0.20	3.00	5.00	0.06
Soil depth	5.00	4.00	1.00	3.00	2.00	0.33	0.50	6.00	7.00	0.15
Soil texture	3.00	2.00	0.33	1.00	0.50	0.20	0.25	4.00	5.00	0.08
Soil drainage	4.00	3.00	0.50	2.00	1.00	0.25	0.33	5.00	7.00	0.11
Rainfall	7.00	6.00	3.00	5.00	4.00	1.00	2.00	8.00	9.00	0.31
Temperature	6.00	5.00	2.00	4.00	3.00	0.50	1.00	7.00	8.00	0.22
Soil pH	0.50	0.33	0.17	0.25	0.20	0.13	0.14	1.00	2.00	0.03
SOC	0.33	0.20	0.14	0.20	0.14	0.11	0.13	0.50	1.00	0.02

Consistency index (CI) = $({\lambda }_{max}-n )/(n-1)$  = 0.1034.

Number of parameters (n) = 9.

Maximum Eigenvalue $${(\lambda }_{\text{max}})= 9.8274$$.

Random index (RI) = 1.46.

Consistency ratio (CR) = CI/RI = 0.0708.

## Results

### Topological parameters

#### Elevation

**Fig. 3:**
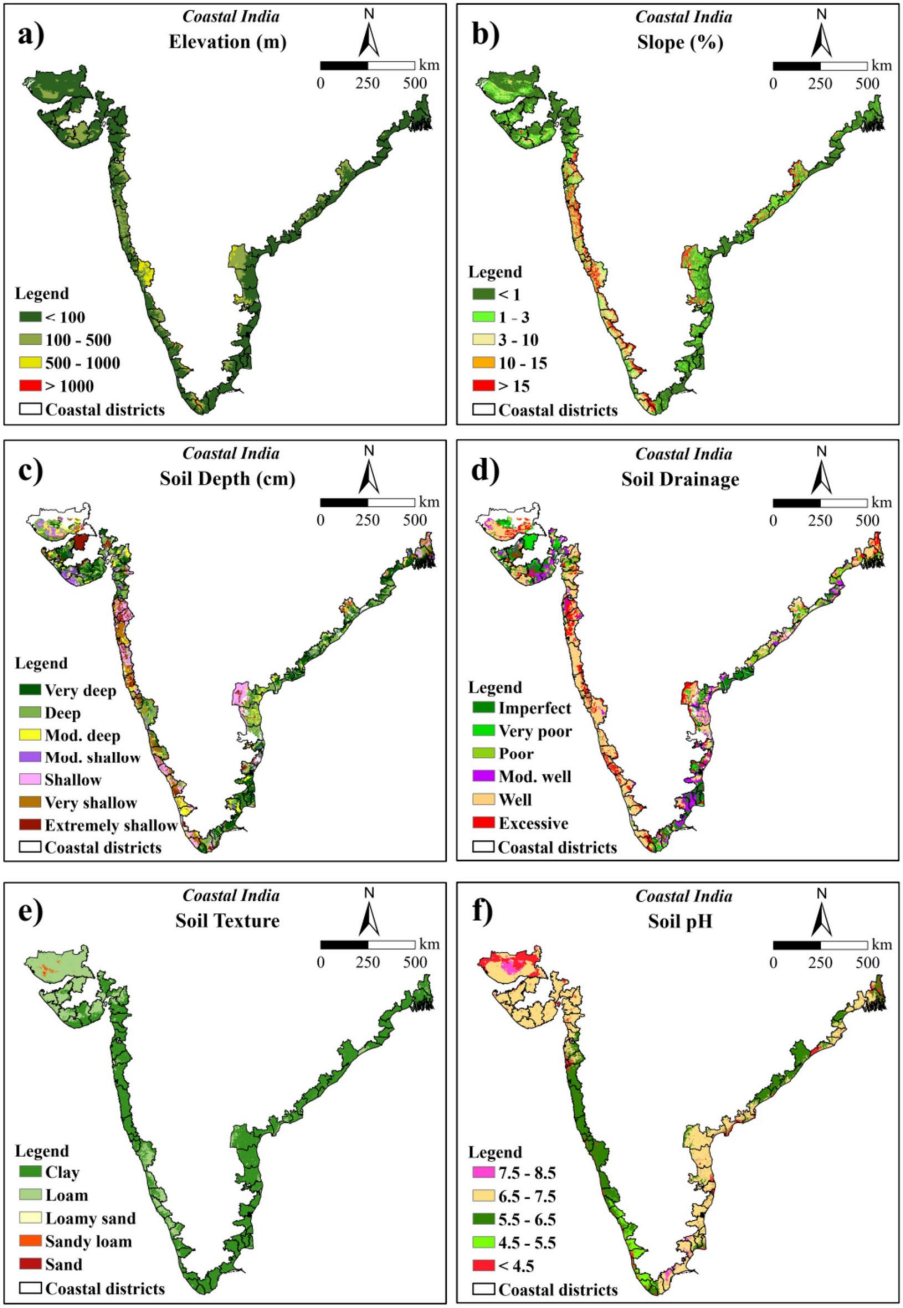

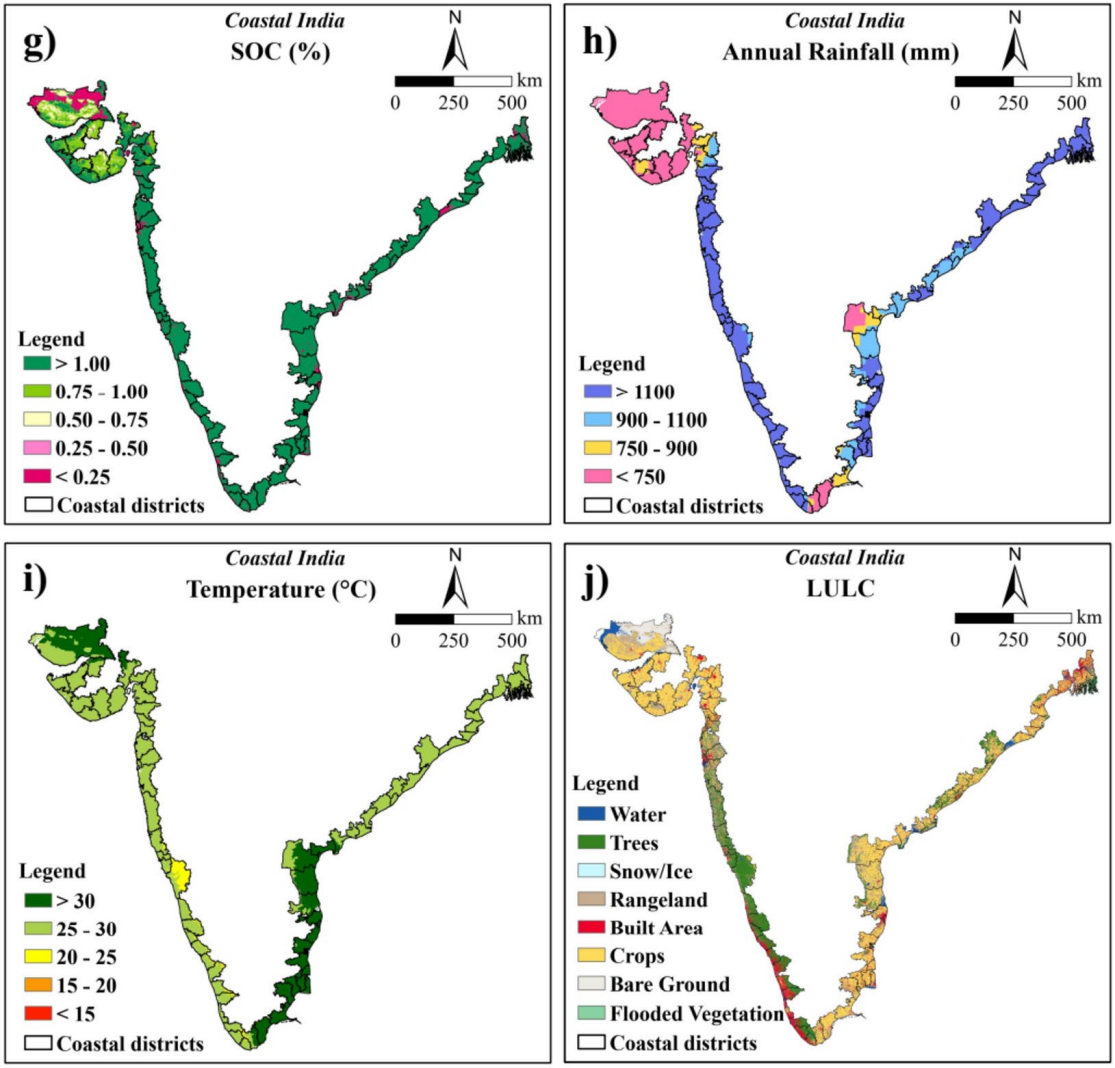
Reclassified maps used for rice suitability analysis (**a**) elevation, (**b**) slope, (**c**) soil depth, (**d**) soil drainage, (**e**) soil texture, (**f**) soil pH, (**g**) SOC, (**h**) annual rainfall, (**i**) temperature and (**j**) LULC (The maps were generated using ArcGIS software version 9.1 https://www.arcgis.com/index.html).

In this study, elevation and slope were regarded as significant topological parameters for rice cultivation. Based on SRTM data, the elevation of the coastal Indian region ranged from − 5 to 2500 m above the Mean Sea Level (MSL). Regarding rice cultivation, elevation was reclassified into four major categories which were < 100 m, 100–500 m, 500–1000 m, and > 1000 m. Rice is traditionally cultivated in lowlands where water stagnation is favoured, making elevations below 100 m preferable for its growth^45,46^. The elevation with < 100 m class covered about 73.08% of the total study area. The regions with 100–500 m elevation accounted for 22.46% which may be used for rice cultivation. The remaining regions with elevations 500–1000 and > 1000 m covered about 3.81 and 0.65% of the study area, respectively, which are not suitable for producing rice. Higher weights were allotted to the areas with < 100 m elevation (Fig. 3a and Table 5).

**Table 5:** Rice parameter sub-classes, the area under each sub-class and weights.

Parameter	Class	Area (ha)	Area (%)	Weights
Elevation (m)	< 100	24,029,805.11	73.08	0.58
	100–500	7,386,142.61	22.46	0.26
	500–1000	1,253,311.36	3.81	0.12
	> 1000	214,111.36	0.65	0.05
n = 4; λ_max_ = 4.140; CI = 0.047; RI = 0.900; CR = 0.052				
Slope (%)	Level to nearly level (< 1)	14,813,367.84	45.05	0.51
	Very gentle slope (1–3)	8,903,905.34	27.08	0.24
	Gentle slope (3–10)	4,640,617.84	14.11	0.14
	Moderate slope (10–15)	1,391,961.59	4.23	0.06
	Moderate steep (> 15)	3,133,517.84	9.53	0.04
n = 5; λ_max_ = 5.326; CI = 0.081; RI = 1.12; CR = 0.073				
Depth (cm)	Very Deep (> 125)	7,889,766.32	23.99	0.28
	Deep (100–125)	8,670,316.32	26.37	0.28
	Mod. deep (75–100)	3,698,547.57	11.25	0.20
	Mod. shallow (50–75)	2,021,210.07	6.15	0.12
	Shallow (25–50)	4,220,660.07	12.84	0.06
	Very shallow (10–25)	3,470,272.57	10.55	0.03
	Extremely shallow (< 10)	2,912,597.57	8.86	0.02
n = 7; λ_max_ = 7.409; CI = 0.068; RI = 1.32; CR = 0.052				
Texture	Clayey	21,911,785.34	66.63	0.52
	Loam	10,812,841.59	32.88	0.22
	Sandy loam	158,529.09	0.48	0.11
	Loamy sand	147.84	0.00	0.10
	Sand	66.59	0.00	0.04
n = 5; λ_max_ = 5.108; CI = 0.027; RI = 1.12; CR = 0.024				
Drainage	Imperfect	5,488,608.62	16.69	0.39
	Very poor	2,298,271.12	6.99	0.28
	Poor	2,860,714.87	8.70	0.18
	Moderately well drained	4,268,464.87	12.98	0.08
	Well drained	13,206,446.12	40.16	0.05
	Excessive	4,760,864.87	14.48	0.03
n = 6; λ_max_ = 6.293; CI = 0.059; RI = 1.24; CR = 0.047				
SOC (%)	Very high (> 1.00)	24,744,391.59	75.25	0.43
	High (0.75–1.00)	3,677,329.09	11.18	0.31
	Medium (0.50–0.75)	1,216,172.84	3.70	0.14
	Low (0.25–0.50)	165,397.84	0.50	0.07
	Very low (< 0.25)	3,080,079.09	9.37	0.04
n = 5; λ_max_ = 5.133; CI = 0.033; RI = 1.12; CR = 0.030				
pH	Strongly acidic (< 4.5)	3,851,085.99	11.71	0.09
	Moderately acidic (4.5–5.5)	1,776,889.87	5.40	0.20
	Slightly acidic (5.5–6.5)	9,055,183.62	27.54	0.46
	Neutral (6.5–7.5)	17,411,514.87	52.95	0.20
	Slightly alkaline (7.5–8.5)	788,696.12	2.40	0.04
n = 5; λ_max_ = 5.222; CI = 0.055; RI = 1.12; CR = 0.049				
Rainfall (mm)	> 1100	15,506,442.61	47.16	0.54
	900–1100	4,662,567.61	14.18	0.29
	750–900	2,419,373.86	7.36	0.11
	< 750	10,294,986.36	31.31	0.06
n = 4; λ_max_ = 4.009; CI = 0.003; RI = 0.900; CR = 0.003				
Temperature (°C)	> 30	9,723,526.59	29.57	0.48
	25–30	21,502,207.84	65.39	0.27
	20–25	1,439,082.84	4.38	0.15
	15–20	124,176.59	0.38	0.06
	< 15	94,376.59	0.29	0.04
n = 5; λ_max_ = 5.239; CI = 0.060; RI = 1.12; CR = 0.053				

**Fig. 4:**
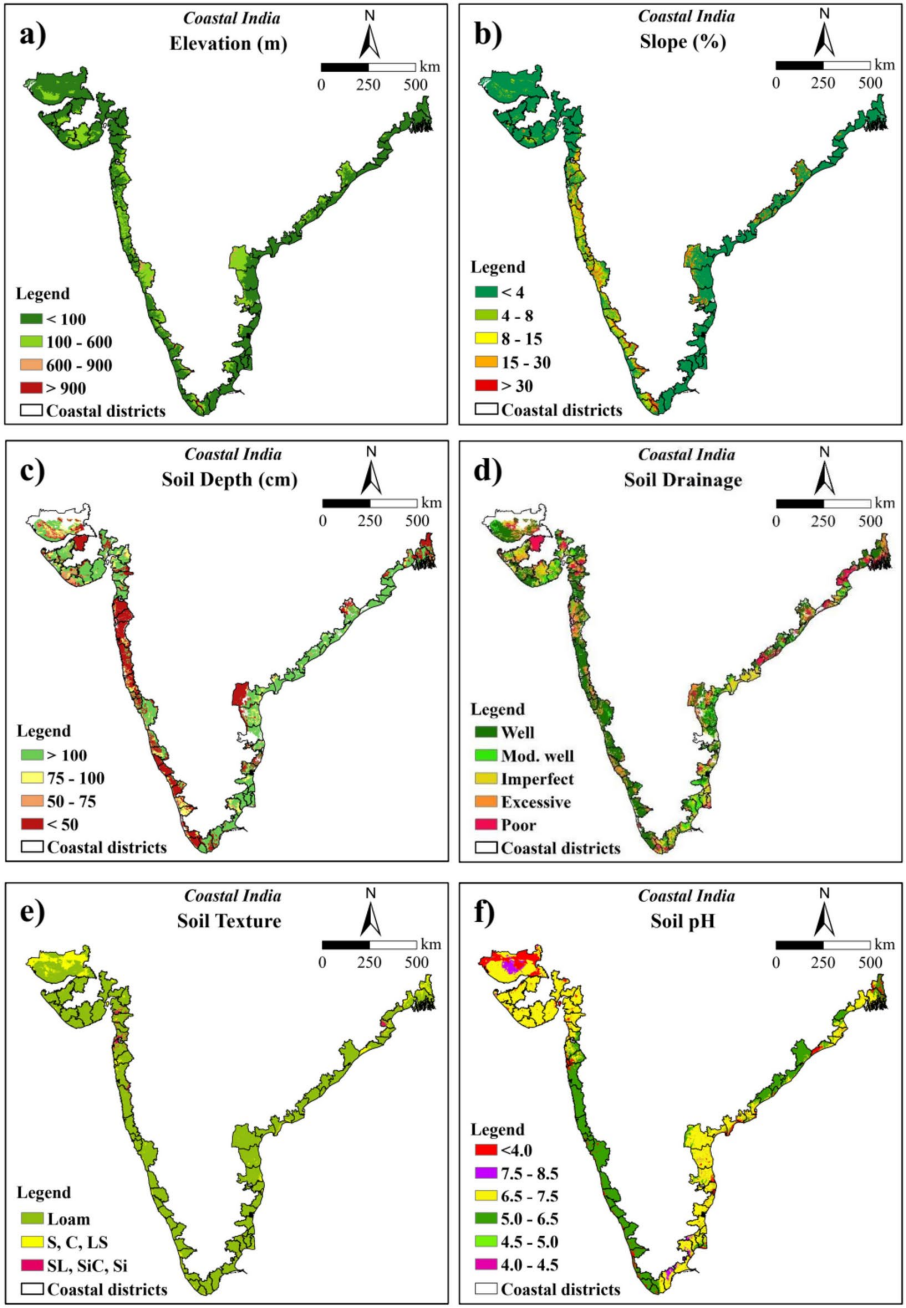

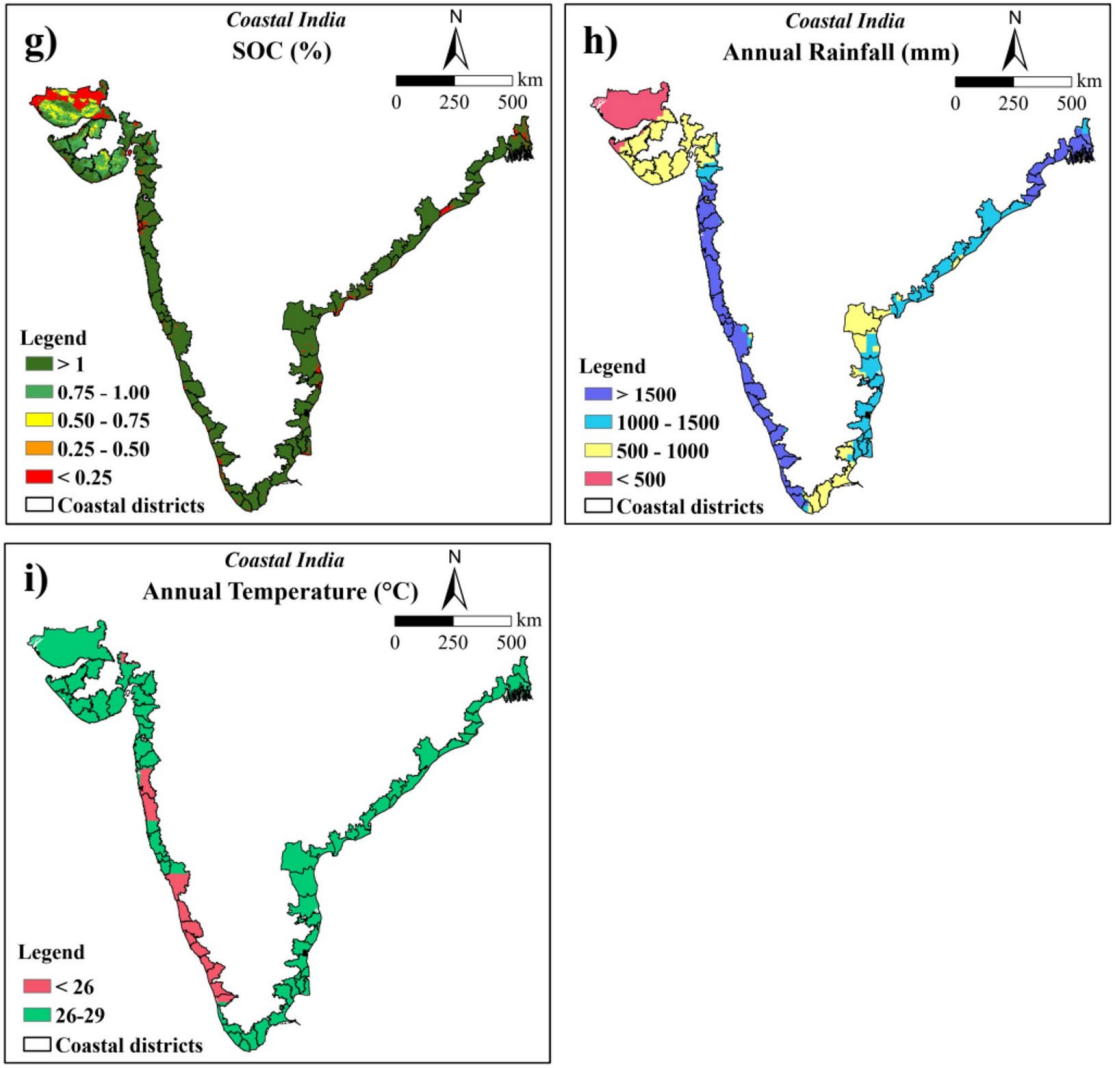
Reclassified maps used for coconut suitability analysis (**a**) elevation, (**b**) slope, (**c**) soil depth, (**d**) soil drainage, (**e**) soil texture, (**f**) soil pH, (**g**) SOC, (**h**) annual rainfall, and (**i**) temperature (The maps were generated using ArcGIS software version 9.1 https://www.arcgis.com/index.html).

For the production of coconut, the elevation was classified into 4 classes based on its suitable conditions namely, < 100, 100–600, 600–900, and > 900 m. Coconut can flourish well up to an elevation of 600 m above MSL. It can also be grown in areas with an elevation greater than 600 m, provided the temperature is favourable for its growth^47^. The areas with elevation < 100 m occupied 73.08% of the total study area. The sites with 100–600, 600–900, and > 900 m elevations inhabit 24.46, 1.72, and 0.74% area, respectively. The higher weights were assigned to the areas with < 100 m elevation (Fig. 4a and Table 6).

**Table 6:** Coconut parameter sub-classes, area under each sub-class and weights.

Parameter	Class	Area (ha)	Area (%)	Weights
Elevation (m)	< 100	24,029,805.11	73.08	0.58
	100–600	8,044,155.11	24.46	0.26
	600–900	564,998.86	1.72	0.12
	> 900	244,411.36	0.74	0.05
n = 4; λ_max_ = 4.140; CI = 0.047; RI = 0.9; CR = 0.052				
Slope (%)	< 4	24,815,907.12	76.49	0.51
	4–8	2,773,563.37	8.05	0.24
	8–15	2,160,382.12	6.15	0.14
	15–30	2,007,737.05	6.02	0.06
	> 30	1,125,780.80	3.29	0.04
n = 5; λ_max_ = 5.326; CI = 0.081; RI = 1.12; CR = 0.073				
Depth (cm)	> 100	16,560,082.63	50.36	0.52
	75–100	3,698,547.57	11.25	0.30
	50–75	2,021,210.07	6.15	0.12
	< 50	10,603,530.20	32.25	0.06
n = 4; λ_max_ = 4.118; CI = 0.039; RI = 0.9; CR = 0.044				
Texture	Loam	29,038,342.24	88.31	0.67
	SL, SiC, Si	3,359,098.49	10.22	0.24
	S, C, LS	485,929.74	1.48	0.09
n = 3; λ_max_ = 3.014; CI = 0.007; RI = 0.58; CR = 0.012				
Drainage	Well drained	13,206,446.12	40.16	0.49
	Moderately well drained	4,268,464.87	12.98	0.24
	Imperfect	5,488,608.62	16.69	0.12
	Excessive	4,760,864.87	14.48	0.10
	Poorly drained	5,158,985.99	15.69	0.04
n = 5; λ_max_ = 5.371; CI = 0.093; RI = 1.12; CR = 0.083				
SOC (%)	> 1.00	24,744,391.59	75.25	0.43
	0.75–1.00	3,677,329.09	11.18	0.31
	0.50–0.75	1,216,172.84	3.70	0.14
	0.25–0.50	165,397.84	0.50	0.07
	< 0.25	3,080,079.09	9.37	0.04
n = 5; λ_max_ = 5.133; CI = 0.033; RI = 1.12; CR = 0.030				
pH	5.0–6.5	10,717,927.37	32.59	0.57
	6.5–7.5, 4.5–5.0	17,525,660.99	53.2	0.27
	7.5–8.5, 4.0–4.5	895,704.74	2.73	0.11
	< 4.0	3,744,077.37	11.39	0.05
n = 4; λ_max_ = 4.191; CI = 0.064; RI = 0.9; CR = 0.071				
Rainfall (mm)	> 1500	10,094,961.36	30.70	0.54
	1000–1500	8,230,573.86	25.03	0.29
	500–1000	10,333,555.11	31.42	0.11
	< 500	4,224,280.11	12.85	0.06
n = 4; λ_max_ = 4.009; CI = 0.003; RI = 0.9; CR = 0.003				
Temperature (°C)	26–29	28,232,447.73	85.86	0.75
	< 26	4,650,922.73	14.14	0.25
n = 2; λ_max_ = 2.000; CI = 0.00; RI = 0; CR = 0.00				

#### Slope

SRTM data was used to calculate the slope map. A lower slope indicates flatter surfaces while a higher slope represents a sharp and steep slope. The slope of coastal India ranged from 0 to 207.62%. For the cultivation of rice, it was classified into five classes namely < 1, 1–3, 3–10, 10–15 and > 15%^48^ out of which the maximum area comes under level to nearly level slope (< 1%) with 45.05% area. The level to nearly level class was assigned with higher weightage as it allows standing water which is favorable for better growth and development of rice. Further, the very gently sloping (1–3%) class covered 27.08% area. The remaining classes with gently sloping (3–10%), moderately sloping (10–15%) and moderately steep (> 15%) covered about 14.11, 4.23 and 9.53% areas, respectively (Fig. 3b and Table 5).

The slope of the coastal region was classified into five major classes based on the requirement of a coconut, viz. < 4, 4–8, 8–15, 15–30, and > 30% (slightly modified from Naidu et al.^24^). The areas under each of these classes were 76.49, 8.05, 6.15, 6.02, 3.29% of the total study region, respectively. For coconut cultivation, higher weights were allotted to the class with < 4% slope which was appropriate for its growth, since coconut prefers low-lying areas and higher slopes can affect the plant growth through erosion of fertile soil layer with surface runoff (Fig. 4b and Table 6).

### Pedological parameters

#### Soil depth

In the present study, soil depth for rice was categorized into seven classes ranging from extremely shallow (< 10 cm) to very deep (> 125 cm) soil depths. The majority area of the study region is very deep (> 125 cm) covering about 23.99% followed by deep (100–125 cm) soil depths with 26.37% area. The remaining classes namely moderately deep (75–100 cm), moderately shallow (50–75 cm), shallow (25–50 cm), very shallow (10–25 cm), and extremely shallow (< 10 cm) soil depths covered 11.25, 6.15, 12.84, 10.55 and 8.86% of the total area, respectively. The higher weights were assigned to very deep soils followed by deep soil depths since higher depths will permit better root growth. Better root growth will help to extract higher soil moisture and nutrients leading to better crop growth and ultimately higher yield (Fig. 3c and Table 5).

Soil depth data for coconut growing conditions was reclassified into 4 classes, namely, > 100, 75–100, 50–75, and < 50 cm^24^ with 50.36, 11.25, 6.15, and 32.25% of the area belonged to each of these classes, respectively. Soil depth > 100 cm was considered to be most suitable for coconut and was assigned the highest weights among others (Fig. 4c and Table 6).

#### Soil drainage

Different soil characteristics such as texture, structure and porosity of soil affect the soil drainage parameter. In the current study, soil drainage for rice was classified into six classes namely imperfect, very poor, poor, moderately well-drained, well-drained and excessive^11^. The areas in each of these classes were 16.69, 6.99, 8.70, 12.98, 40.16 and 14.48%, respectively. The maximum weightage was assigned to imperfect drainage because rice requires standing water for its growth and development. Imperfectly drained soil will allow standing water for a longer period (Fig. 3d and Table 5).

Soil drainage for coconut production was reclassified into 5 major classes namely, well-drained, moderately well-drained, imperfect, excessive, and poorly drained^11^. These classes represented 40.16, 12.98, 16.69, 14.48, and 15.69% of the total study area, respectively. Coconut prefers well-drained soils for their optimal growth and development; hence this type of drainage system was allotted the highest weight followed by other sub-classes (Fig. 4d and Table 6).

#### Soil texture

Soil texture is one of the important factors responsible for crop production. The circulation and availability of air and water in the soil, root growth, water and nutrient intake, are mostly influenced by soil texture. For rice suitability, soil texture was classified into five classes viz*.* clayey (consisting of clay, silty clay, clay loam, silty clay loam, sandy clay), loam (consisting of sandy clay loam, silt loam, loam, silt), loamy sand, sandy loam and sand^24^ where clayey soil texture was assigned with the highest weights. Because clayey soil can hold more water and nutrients than any other soil type, giving optimal growing conditions for rice crops. The majority areas of the study region were under clayey soils (21,911,785.34 ha) followed by loam (10,812,841.59 ha), sandy loam (158,529.09 ha), loamy sand (147.84 ha) and sand (66.59 ha) (Fig. 3e and Table 5).

For coconut, soil texture present in the study region was reclassified into three classes namely, 1) loam (comprising of clay loam, loam, sandy clay loam, sandy clay, silty clay loam, silty loam); 2) sandy loam (SL), silty clay (SiC), silt (Si); 3) sand (S), clay (C), and loamy sand (LS)^24^. The classes are composed of 88.31, 10.22, and 1.48% of the total area. Loam soil type predominated in the study region and was assigned the highest weight due to its suitability for coconut cultivation (Fig. 4e and Table 6).

#### Soil pH

Soil pH is a critical factor influencing crop growth. Several chemical and biological processes inside the soil are regulated and controlled by soil pH. The increase in soil pH leads to a decrease in shoot weight and panicle number in rice, resulting in a significant decline in rice yield^49^. Based on the suitable pH range for rice, it was categorized into five classes namely strongly acidic (< 4.5), moderately acidic (4.5–5.5), slightly acidic (5.5–6.5), neutral (6.5–7.5) and slightly alkaline (7.5–8.5)^24^. Among these classes, the coastal region predominantly consisted of neutral pH covering about 52.95% (17,411,514.87 ha). Slightly acidic soils were mainly observed in the eastern and western parts of the study region covering 27.54% of the total area. Slightly alkaline, moderately acidic and strongly acidic soils were observed more in the western parts than eastern parts of the study region. The areas covered by these classes were 2.40, 5.40 and 11.71%, of the total area respectively. The slightly acidic soil was given greater importance due to its pH level falling within the optimal range of 5.5 to 6.5 for rice cultivation. Neutral and moderately acidic soils, which are also suitable for rice development, were assigned subsequently lower weights (Fig. 3f and Table 5).

The optimal soil pH range for coconut plantations is typically between 5.0 and 6.5. Based on this requirement, the pH values were categorized into six major classes viz*.*, < 4.0, 4.0–4.5, 4.5–5.0, 5.0–6.5, 6.5–7.5 and 7.5–8.5^24^. Areas with pH less than 4.0 covered 11.39%, pH 4.0–4.5 covered 0.33%, pH 4.5–5.0 covered 0.35%, the optimal pH range of 5.0–6.5 covered 32.59%, pH 6.5–7.5 covered 52.95%, and pH 7.5–8.5 covered 2.40%. The highest weights were assigned to the pH values ranging between 5.0 and 6.5 for coconut suitability. Following this, 6.5–7.5 and 4.5–5.0, and 7.5–8.5 and 4.0–4.5 were assigned similar weights. Lastly, pH values below 4.0 received the lowest weight with respect to coconut suitability (Fig. 4f and Table 6).

#### SOC

The higher SOC content in the soil improves the growth of rice by supplying the required amount of nutrients. It is also essential for a variety of soil activities and ecological characteristics. The SOC under anaerobic waterlogged conditions decomposes more slowly than the upland soils^50^. The SOC content for both rice and coconut were categorized into five groups: < 0.25%, 0.25–0.50%, 0.50–0.75%, 0.75–1.00% and > 1.00% (extended classification adopted from Singh et al.^51^). The majority of the study region had a SOC > 1.00%, covering 75.25% (24,744,391.59 ha) of the total area. This was followed by 0.75–1.00, < 0.25, 0.50–0.75 and 0.25–0.50 classes with 11.18, 9.37, 3.70 and 0.50% of the total area, respectively (Figs. 3g, 4g and Tables 5, 6). Greater weights were assigned to higher SOC content as higher SOC levels indicate better soil quality.

### Climatic parameters

#### Annual rainfall

Rainfall is a crucial factor for rice cultivation due to its high-water requirements compared to other crops. Along the coastal region, the mean annual rainfall varied from 350.09 to 5043.19 mm. The optimal rainfall range for rice cultivation was categorized into four classes: < 750 mm, covering 31.31% of the area; 750–900 mm, covering 7.36%; 900–1100 mm, covering 14.18%; and greater than 1100 mm, covering 47.16%. Greater weightage was given to annual rainfall > 1100 mm, aligning with the optimal range of 1110–1250 mm for rice cultivation (Fig. 3h and Table 5).

The ideal range of mean annual rainfall necessary for a coconut plant’s effective growth and productivity is between 1500 and 2500 mm. Rainfall for coconuts was classified into four categories: > 1500, 1000–1500, 500–1000, and < 500 mm^24^. The area calculated corresponding to each category was 30.70, 25.03, 31.42 and 12.85% out of the total study area, respectively. The subclass with a higher amount of rainfall, that is > 1500 mm, received the highest weightage, followed by subsequent classes (Fig. 4h and Table 6).

#### Temperature

Temperature is another critical factor influencing the growth of rice. It requires a greater temperature range (30–34 °C) to sustain. For rice production, the temperature of the coastal region was determined considering the length of its growth period (June, July, August and September). The temperature during the rice growing season in the study region ranged from 13.30 to 31.72 °C. It was categorized into five classes: < 15, 15–20, 20–25, 25–30 and > 30 °C amongst which temperature with > 30 °C was assigned the highest weight. The area covered across these classes were 0.29, 0.38, 4.38, 65.39and 29.57%, respectively (Fig. 3i and Table 5).

The mean annual temperature was used for coconut suitability analysis which varied between 23.76 and 28.90 °C in the study region. Suitable areas for coconut growth and development significantly depend on the surrounding temperature where the plant is grown. Given its tropical nature, it requires high temperature throughout the year with high humidity in extreme summer periods. Consequently, the coastal region of India is highly suitable for coconut cultivation. Temperature in coastal India was classified into two sub-classes: < 26 and 26–29 °C^24^. A higher weight was attributed to temperatures ranging from 26 to 29 °C. Each of these groups covers an area of 85.86% (28,232,447.73 ha) and 14.14% (4,650,922.73 ha), respectively (Fig. 4i and Table 6).

This LULC map containing eight classes: water, trees, flooded vegetation, crops, built area, bare ground, snow/ice and rangeland, was used for extracting the suitable areas for rice and coconut cultivation (Fig. 3j).

### Crop suitability analysis for rice and coconut using AHP

Based on the AHP analysis conducted for rice, rainfall had the highest weightage (0.29) followed by, soil drainage (0.25), soil texture (0.16), and soil depth (0.11). Both slope and elevation were assigned equal weights (0.06) due to the interdependence of slope and elevation. Weights assigned to SOC (0.04), temperature (0.03) and soil pH (0.02) were less than 0.05. Further, CR for rice was computed as the ratio of CI (0.1140) to the random index value for nine parameters (RI = 1.46). The CR value of 0.0781 was below the threshold of 0.1 and hence accepted for rice suitability analysis (Table 3).

For the coconut suitability analysis, the rainfall of the study area received the highest weight of 0.31, followed by temperature (0.22), soil depth (0.15), soil drainage (0.11), soil texture (0.08), slope (0.06), elevation (0.04), pH (0.03), and SOC (0.02) as per AHP. The calculated CI for coconut was 0.1034 and the total number of parameters corresponding to this matrix was nine (n = 9). Based on these two values, the CR value was computed, which was found to be 0.0708. Since the CR was less than 0.1, it was accepted (Table 4).

Similarly, using the AHP approach, each sub-class for all nine parameters of rice and coconut was assigned weights. The higher weightage was allotted to the best suitable sub-class with lesser weights were given to subsequent sub-classes and CR value was calculated (Supplementary Table 1 and 2).

**Fig. 5:**
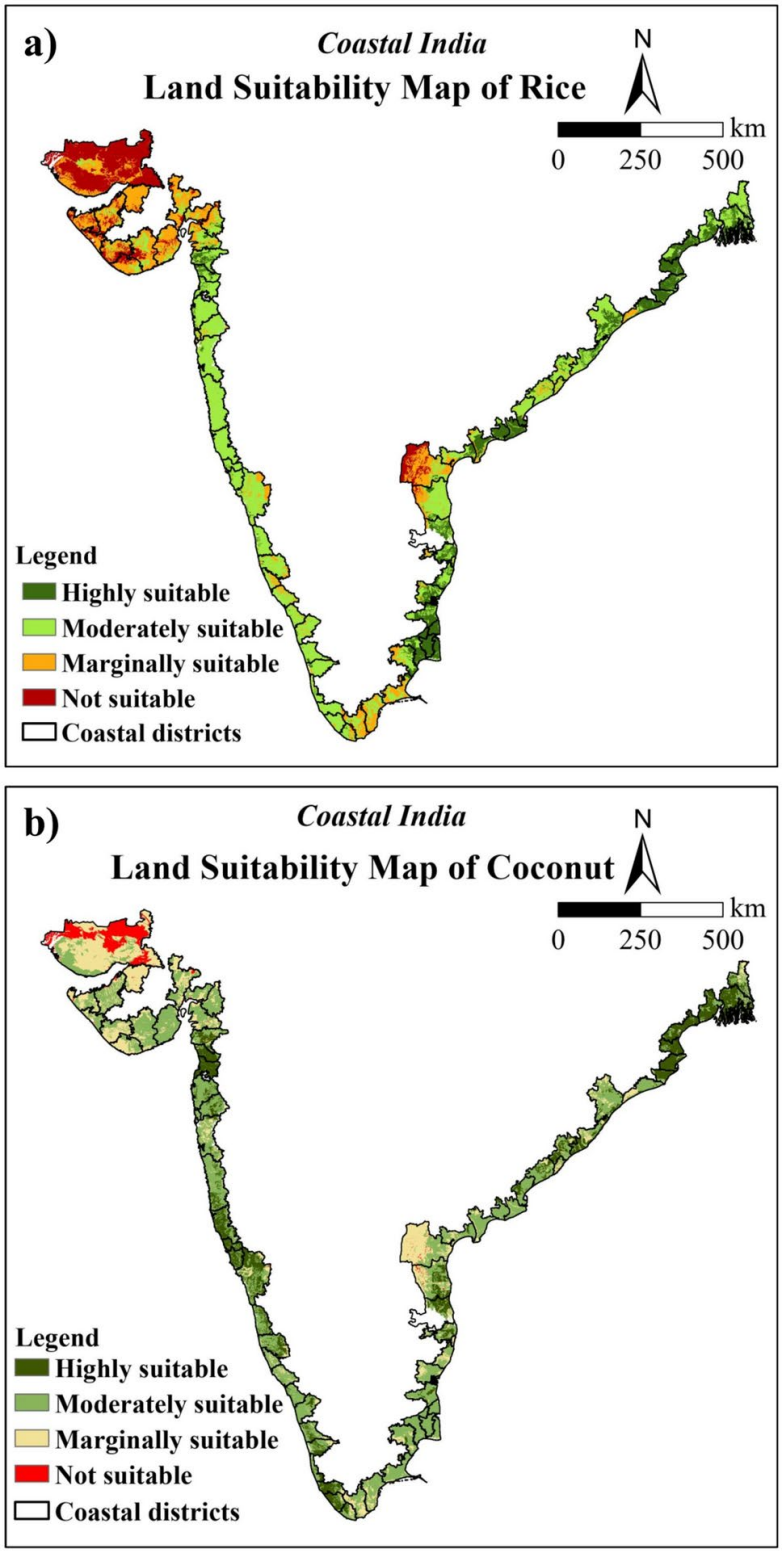
Crop suitability maps of coastal region of India for :(a) rice, and (b) coconut (The maps were generated using ArcGIS software version 9.1 https://www.arcgis.com/index.html).

The crop suitability map was produced based on the 9 factors affecting the rice and coconut growth using the weighted overlay analysis method by considering the AHP calculated weights. The crop suitability index for rice ranged from 0.072 to 0.466 while for coconut, it ranged from 0.145 to 0.545 with higher values indicating better suitability. The derived crop suitability maps were reclassified into four major suitability classes using equal intervals namely highly suitable (S1), moderately suitable (S2), marginally suitable (S3), and not suitable areas (N)**.** Area suitable for rice crop under each of these classes was 16.10, 44.06, 24.11, and 15.73%, respectively (Fig. 5a and Table 7). The corresponding areas suitable for coconut were 18.74, 50.40, 25.25, and 5.60%, respectively (Fig. 5b and Table 8).

**Table 7:** Area suitable for rice cultivation in coastal region before and after masking of non-cropland area.

Class	Before masking		After masking	
	Area (ha)	Area (%)	Area (ha)	Area (%)
Highly suitable	5,293,386.4	16.10	4,498,035.4	13.68
Moderately suitable	14,489,161.4	44.06	6,333,154.1	19.26
Marginally suitable	7,927,948.9	24.11	6,034,816.6	18.35
Not suitable	5,172,873.9	15.73	4,524,885.4	13.76
Non-cropland area	–	–	11,492,479.0	34.95
Total	32,883,370.5	100	32,883,370.5	100

**Table 8:** Area suitable for coconut cultivation in coastal region before and after masking of non-cropland area.

Class	Before masking		After masking	
	Area (ha)	Area (%)	Area (ha)	Area (%)
Highly suitable	6,163,267.6	18.74	3,617,660.4	11.00
Moderately suitable	16,574,723.9	50.40	9,010,122.9	27.40
Marginally suitable	8,304,430.1	25.25	6,031,010.4	18.34
Not suitable	1,840,948.9	5.60	2,732,097.9	8.31
Non-cropland	–	–	11,492,479.0	34.95
Total area	32,883,370.5	100	32,883,370.5	100

Taking into consideration the land available for agriculture, the LULC map was reclassified into two classes i.e., cropland area (flooded vegetation, crops, bare ground, rangeland) and non-cropland area (water, trees, built area, and snow/ice) which was used to generate crop mask. The observed areas corresponding to these cropland and non-cropland regions were 65.05 and 34.95%, respectively (Supplementary Table 3).

**Fig. 6:**
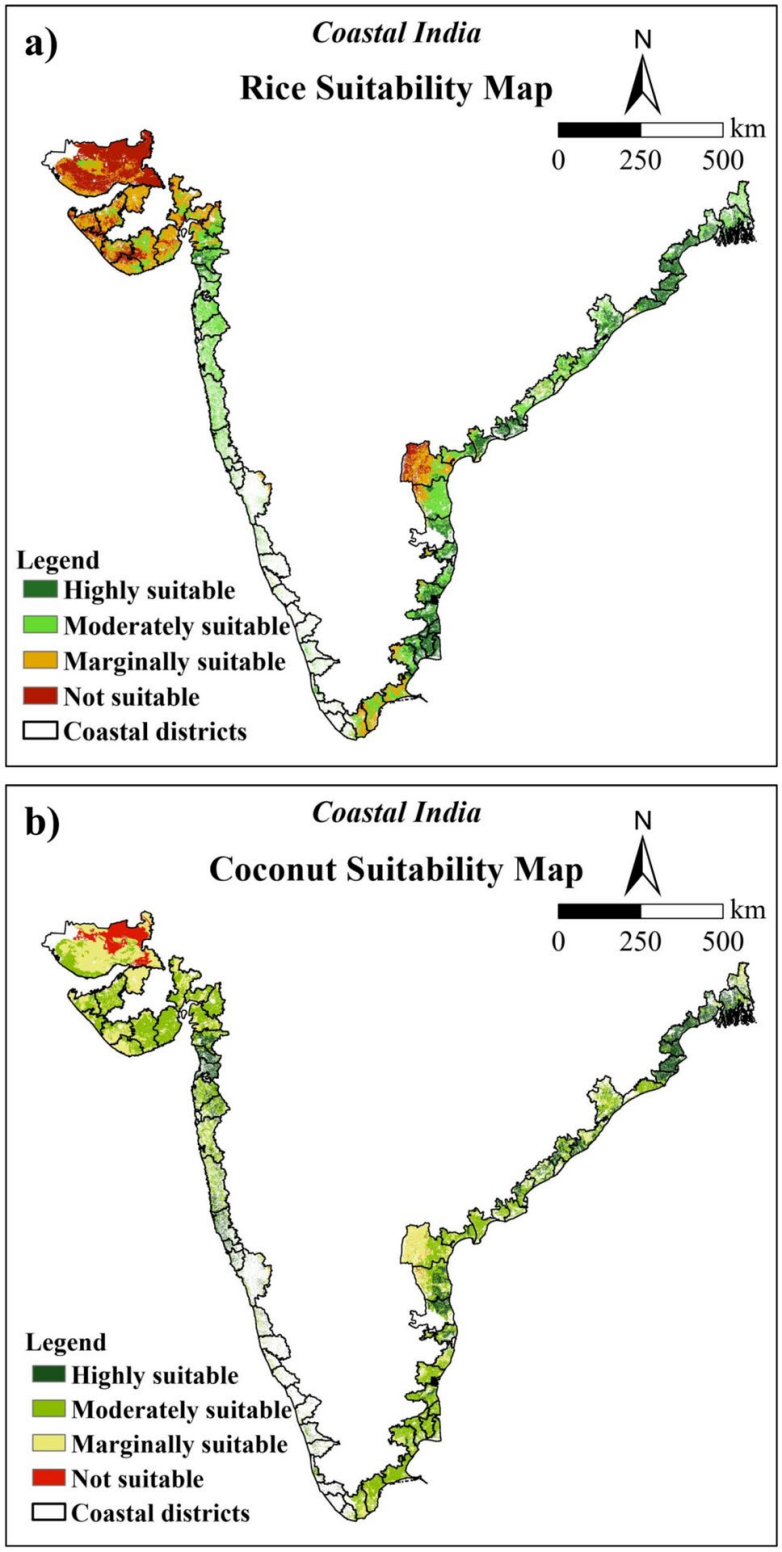
Crop suitability maps of the coastal region of India for (**a**) rice, and (**b**) coconut (after exclusion of non-cropland area) (The maps were generated using ArcGIS software version 9.1 https://www.arcgis.com/index.html).

The crop mask was used to extract rice and coconut suitable areas. Within the study region, 13.68% of area was found highly suitable for rice cultivation, while 19.26% was moderately suitable, 18.35% was marginally suitable and 13.76% was not suitable (Fig. 6a and Table 7). Concurrently with rice, areas suitable for coconut were 11.00, 27.40, 18.34 and 8.31% of the total study region (Fig. 6b and Table 8). Around 34.95% area of coastal India is permanently not suitable for crop cultivation as it includes the non-cropland (water, trees, built area, and snow/ice) areas.

**Fig. 7:**
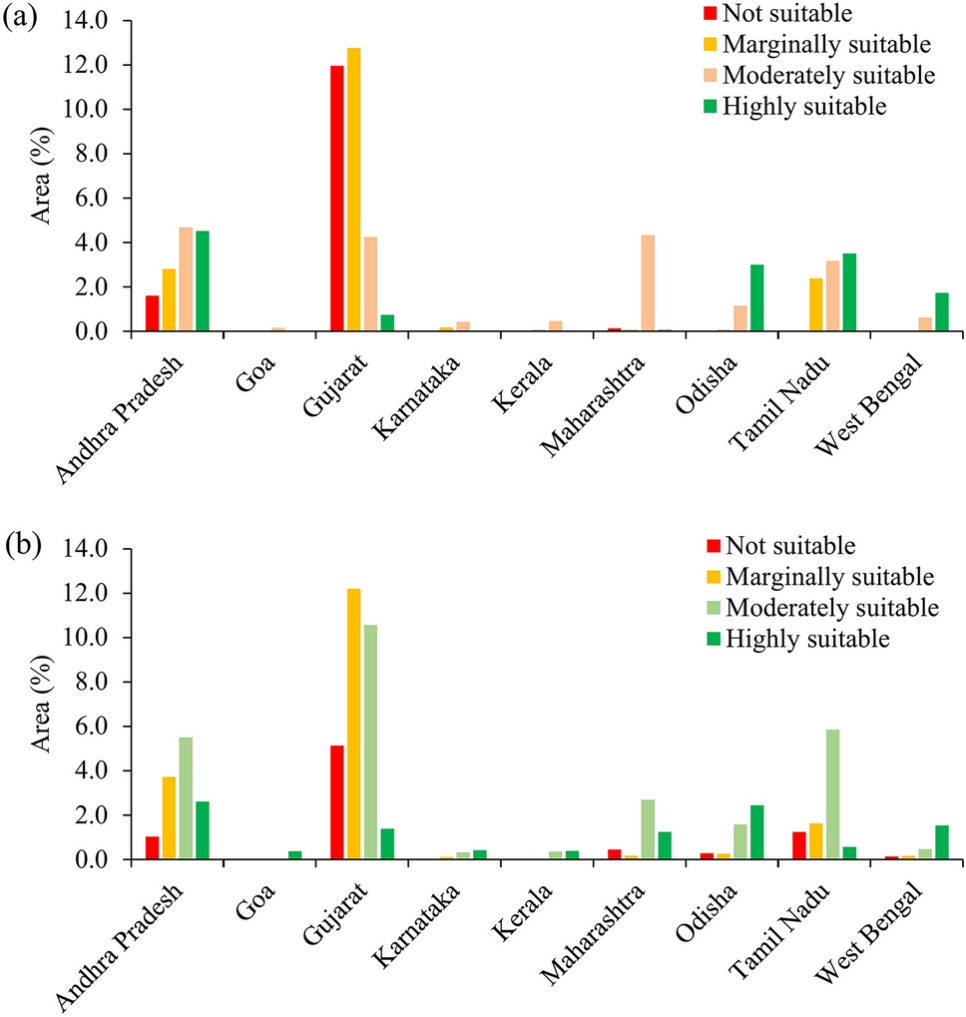
State-wise area suitable for (a) rice and (b) coconut in coastal region after masking of non-cropland area.

The findings revealed that highly suitable areas for rice were mostly distributed on the eastern coast of India with 78.71% area than the western coast with 21.29%. Among the states covered, highly suitable area for rice cultivation was found in Andhra Pradesh (4.51%) followed by Tamil Nadu (3.51%) and Odisha (3.00%) (Fig. 7a). A limited number of districts in Gujarat, Maharashtra, Goa, Karnataka, and Kerala exhibited sparsely distributed highly suitable areas for rice cultivation. It was discovered that coastal areas in eastern India particularly Andhra Pradesh (2.61%) and Odisha (2.45%) were better suited for coconut than those in western India (Fig. 7b). Moderately suitable sites for rice account for about 19.26% of the total area, with 36.31% and 63.69% of that area falling under India’s western and eastern coasts, respectively, predominantly in Andhra Pradesh (4.67%) and Maharashtra (4.33%). Moderately suitable regions for coconut were majorly found in the eastern coast except for some districts of Odisha. On the west coast, moderately suitable coconut sites were mostly present in districts of Maharashtra (2.70%) and Gujarat (10.56%), with very few areas in the remaining districts.

For rice, marginally suitable areas account for around 18.35% of the total study region. The eastern and western coastal regions account for about 32.12% and 67.88% of the total marginally suitable areas, respectively. A greater quantity of marginally suitable areas was found on the west coast out of which Gujarat districts had the majority of marginally suitable sites (12.76%). Along the eastern coast, Prakasam, SPSN districts of Andhra Pradesh and some districts of Tamil Nadu had marginally suitable sites. Similarly for coconut, Gujarat had the highest percentage of marginally suitable lands (12.20%) mainly due to low rainfall followed by Andhra Pradesh (3.73%).

The non-suitable areas were determined by geomorphological features such as high slopes and elevations, the presence of bare rocks, drainage requirements, unfavorable temperature, and rainfall. After crop mask, the non-suitable area for rice crops was ~ 13.76% of the total study area. Approximately 19.75% of the unsuitable area is located on the eastern coast of India, while the remaining 80.25% is located on the western coast. On the east coast, some districts of Andhra Pradesh and Tamil Nadu showed non-suitable areas for rice cultivation. The majority of the non-suitable areas in the western coastal region were located in the districts of Gujarat (11.96%) except for Valsad, Surat and Navsari (Fig. 7a). In the west coast, all districts of Gujarat (5.13%) except Valsad and Navsari were found to be unsuitable for coconut production, whilst in the east coast, districts in Andhra Pradesh and Tamil Nadu comprised the majority of the unsuitable areas.

### Validation

**Fig. 8:**
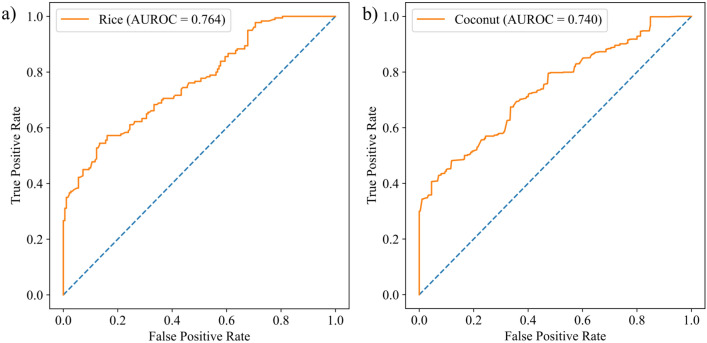
Receiver operating characteristic (ROC) curve with AUROC values for (**a**) rice and (**b**) coconut.

**Fig. 9:**
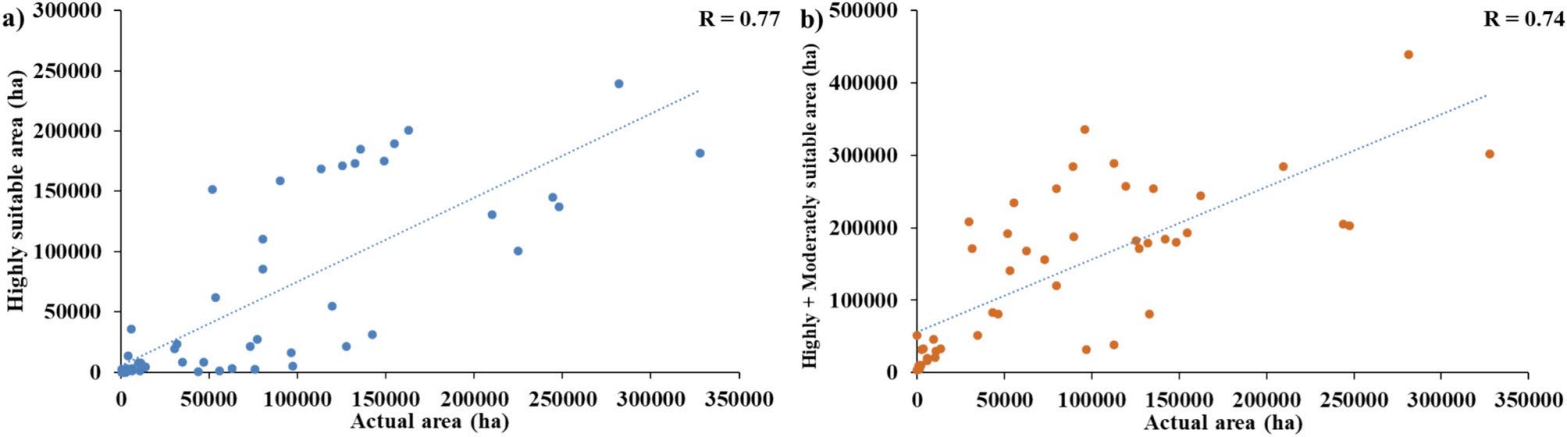
Relationship between (a) actual area vs highly suitable area and (b) actual area vs highly + moderately suitable area for rice.

The area under receiver operating characteristic curve (AUROC) for rice and coconut is presented in Fig. 8. The AUROC values for both rice and coconut were more than 0.7 indicating high accuracy of the suitability maps. Further, the actual area under rice cultivation acquired from the Directorate of Economics & Statistics, Ministry of Agriculture and Farmers Welfare, Government of India, New Delhi for the year 2019–2020, was correlated (R) with district-wise highly suitable areas and highly + moderately suitable areas. For both rice and coconut, the R value for each category was computed. The findings showed a strong association (R = 0.77 and 0.74, respectively) between areas under rice with highly and highly + moderately suitable areas (Fig. 9a,b). However, the correlation for coconut was weak, with R = 0.35 and 0.58 for locations that were highly and highly + moderately suited, respectively (data not provided).

## Discussion

### Climate parameters

In India, monsoon rainfall and rice production are closely related. The annual rainfall in the study region varied between 350.09 and 5043.19 mm. Repetitive floods occurring due to heavy rainfall have greatly affected rice-growing areas and its production. Higher rainfall can cause a reduction in pollination rate and lodging which harms grain quality^52^. On the other hand, severe drought conditions driven by insufficient rainfall can reduce rice yield. The steady and well-distributed rainfall pattern which reduces the likelihood of flood and drought conditions, is best suited for effective rice production^53–55^. Annual rainfall of 1000–2500 mm distributed throughout the year with a good amount of sunlight is best suitable for healthy coconut growth and higher yields. Extreme rainfall events can damage its healthy growth. The severe drought conditions and less amount of rainfall can cause abortion in coconut spadix and inflorescence at the earlier stage of development. It may also reduce female flowers and may cause immature nut falls with reduced nut size. Heavy rains reduce the sunlight and temperature with increased humidity leading to increased incidence of pests and diseases. The outcomes of AHP analysis suggested that rainfall had a major influence on rice and coconut suitability in the study area. This is in line with earlier studies that emphasized the importance of rainfall for crop suitability analysis ^21,56^.

The mean temperature of the study region during the rice growing period from June to September was 23.76 to 28.91 °C which is ideal for rice cultivation. The optimum temperature required for rice throughout its growing period ranges from 21 to 35 °C^24^. The sudden increase or decrease in temperature beyond optimum value results in negative impact on rice yield. The spikelet sterility, length of growing period, photosynthesis and respiration processes get disturbed, thereby lowering crop productivity^52^. The mean annual temperature for coconut plantation ranged from 23.76 to 28.91 °C aligning with the optimal temperature range for coconuts, which is approximately 27 °C ± 5 °C. Extreme high temperatures > 34 °C, greatly affect coconut growth by decreasing photosynthesis during its reproductive stage, which ultimately causes poor fertilization with a reduced nut set. Higher temperature also leads to drying up of the inflorescence.

### Pedological parameters

This study investigated five soil factors for rice and coconut growth namely soil depth, drainage, texture, soil pH, and SOC. Soil depth in the study region was classified from extremely shallow to very deep. The areas with deeper soils provide better root growth in rice crops than shallower soils, allowing the plants to uptake more water and nutrients. Under drought situations, there is a high possibility that the moisture content in soil with shallow depth will drop below the permanent wilting point. It was reported that coconut having adventitious root system, under good management practices require a maximum of 2 m soil depths. The surface soil depth of 2 m with good moisture, aeration and nutrients, unrestricted root development, and absence of rock or hard substratum permit healthy root growth, eventually leading to better yield^57^.

Soil texture highly impacts rice grain production and yield. The soil drainage, water holding capacity, space within pores, and available nutrients are all influenced by texture. For better growth and development, rice requires clayey soils which have a good water-holding capacity (WHC) and more available nutrients than any other type of soils. Sandy soils have low nutrient and WHC, high aeration, and drainage whereas loamy soils have intermediate characteristics^58^. According to the research conducted by Dou et al.^59^, rice grown in clayey soil, showed a 46% more yield and 25% higher panicle number with a bigger size and density of grains than the rice cultivated in sandy soil. Coconut plants can survive in various soil types such as loamy, laterite, coastal sandy, alluvial, clayey and reclaimed soils of the marshy lowlands provided, they have an adequate drainage system. Although coconut is adapted to various soil types, conventional farming methods are difficult in sandy soil due to the low water retention and soil fertility^60^.

The drainage in the study region ranged between excessive and imperfectly drained soils. As rice requires standing water, the imperfect drainage system is best suitable for its growth. The soil drainage is greatly dependent on which type of soil, the crop is grown. Though rice can be grown well under alternate dry and wet conditions, clayey-type soils allowing standing water is perfect for rice growth. Coconut grow well in light textured soils naturally because the water is well-drained in such soils^57^. Coconut plants cannot withstand standing water and a good drainage can help coconut to grow efficiently.

The optimal pH required for rice cultivation is between 5.5 and 6.5. If the soil pH is equal to or greater than 7.5, and less than 4.5, then rice will not grow well in such soils^24^. Huang et al.^49^ reported that with increasing pH value, rice grain yield decreased drastically. It also impacts the uptake of mineral nutrients thereby affecting rice yield. The optimal pH value required for coconut growth varies from 5.0 to 6.5, and can still be grown in areas with a pH value of 8.0, provided all the suitable conditions are available^57^.

SOC content is affected by several geographical variables, including soil type, texture, terrain, land use, and management practices. A higher percentage of SOC is found in the top layers of the soil than in subsoil layers, this may be because of less litter content in subsoils. Paddy soils in the tropical region have greater SOC content since the presence of organic matter is higher in flooded conditions. The anaerobic processes occurring in waterlogged paddy fields help to maintain SOC content^61^. The greater organic matter in the soil leads to increased rice production. SOC content has equal importance for coconut as required for rice growth. Higher SOC content will boost healthy coconut plant growth.

### Topographical parameters

The elevation in the coastal Indian region ranges from − 5 to 2500.77 m above MSL. The optimum elevation for rice cultivation is less than 100 m. Ramadhani et al.^62^ reported that rice yield was higher in the areas with low elevation and flat terrain while the more elevated and hilly topographical regions resulted in less rice grain production. The temperature in the high-elevation areas is lower which is not suitable for rice growth and development as low temperature during rice growth causes severe spikelet sterility^45^. The topography of the land highly influences the rice distribution pattern, since it is responsible for controlling temperature, soil properties, and sunlight which directly affect rice development. It was observed that paddy fields are more common in flat surfaces and are sparsely distributed at higher altitudes^63,64^. Coconut crops grow very well up to 600 m elevation above MSL. However, it can also be grown at an elevation of 1000 m above MSL with good vegetative growth but with poor nut yield and hence not advisable for commercial yield^47^.

The slope of an area is significantly important for agricultural practices^65^. The slope of the coastal Indian region varied from 0 to 207.62%. Rice requires less than 1% of slope so that water can be accumulated. If the slope is greater than one, the water will run off easily and in the absence of standing water rice cannot be grown. The slope angle controls the pace of erosion, soil depth, and the rate of soil development^66^. Wang et al.^63^ reported that the areas with slopes greater than 24, cannot be used for rice cultivation. The direction of sunrays directly depends upon the slope aspect which impacts crop growth and development. Coconut grown on slopes can result in soil erosion, surface water and nutrient runoff impacting its growth and development. Coconut cultivation on slopes should be preferred with contour terracing since it will help reduce soil erosion on slopes.

### Accuracy assessment

The AUROC value for rice reported in this study was found better than machine learning models like AdaBoost, random forest, extreme gradient boosting and Naïve Bayes while it was inferior to support vector machine (AUROC = 0.804) as reported by Singha et al.^67^. Li et al.^68^ and Ali et al.^69^ reported higher AUROC values (> 0.85) for mapping rice suitability in China and South Asia using MaxEnt model. Similarly, Hebbar et al.^47^ also reported better AUROC value of 0.899 for coconut suitability mapping in India. We could not compare the AUROC values reported in this study using AHP method with previous reports as this is the first study where AUROC was utilized to validate AHP based crop suitability maps of rice and coconut. The observed discrepancy between the actual and predicted areas under rice and coconut cultivation can be attributed to several factors. These may include (1) farmers may lack awareness of the potential suitability of their land for these specific crops, leading to suboptimal land use decisions, (2) land might be allocated to alternative crops that offer better economic returns or align more closely with local agricultural practices and market demands, or (3) problem in validation of suitability maps because of inconsistencies between map outputs and actual field conditions owing to resolution mismatch. In certain parts, the suitability for rice or coconut cultivation was very low but still, the crops were growing efficiently due to better management practices.

### Limitations

1. We have considered only nine factors affecting the growth and production of rice and coconut in our analysis. Inclusion of additional factors could provide more accurate and comprehensive estimates of suitable areas for these crops.
2. We have employed AHP with expert opinion for crop suitability mapping. However, advanced geospatial techniques like machine learning and deep learning may further enhance the accuracy of the results.
3. Expert opinions, while valuable, can be highly subjective. It is recommended to use statistics-based objective weighting methods such as frequency ratio or weight of evidence to reduce bias. These approaches can enhance the reliability of the findings by providing a more data-driven basis for analysis.
4. We have mapped the suitability of rice and coconut at 250 m spatial resolution to make balance between spatial details and computational efficiency. However, it might lead to loss of fine scale variabilities in topography, soil and climate with single pixel representing a mix of crop types or land uses. This may limit their use for smallholder farmers or site-specific precision agriculture. So, future studies should utilize higher-resolution data or employ downscaling methods to achieve crop suitability maps at finer spatial resolution tuned to the need of smallholder farmers.

### Policy implications

The findings of this study have significant implications for agricultural policy and land-use planning in coastal regions, especially under the changing climatic scenarios. The delineated highly and moderately suitable areas for rice and coconut cultivation would enable the policymakers to prioritize targeted interventions like development of irrigation and drainage systems in these regions to enhance productivity. Moreover, extension personnel may educate farmers in these areas on best practices for sustainable cultivation, ensuring optimal use of natural resources while reducing land degradation. Furthermore, coastal areas are facing various climate change-related challenges including rising sea levels, salinity intrusion, and erratic rainfall patterns, all of which can jeopardize crop suitability. Integrating suitability maps with climate projections allows policymakers to anticipate shifts in productive zones and develop adaptation and mitigation strategies, such as introducing salt-tolerant crop varieties or diversifying cropping patterns to include other resilient crops in marginally suitable areas. These findings are also having major implications for land-use planning. Crop suitability data may be aligned with wider development plans to ensure that agricultural growth does not encroach on environmentally sensitive or non-arable regions like forests and wetlands. Additionally, this information may be used to guide land allocation, promoting sustainable agriculture and supporting food security goals in densely populated coastal regions. These strategies, when implemented, can assist to balance the competing needs of economic development, environmental sustainability, and social equity, which are especially important in coastal areas due to climatic fluctuation and population expansion.

## Conclusion

The factors considered for the study were soil depth, drainage, texture, rainfall, temperature, elevation, slope, SOC and soil pH. The LULC map of the study region was used to mask the non-agricultural land from the final crop suitability map. The land suitability analysis for rice crop production revealed that around 13.68% of the total study area was highly suitable whereas, 19.26% was moderately suitable, and 18.35% was marginally suitable for rice. For coconut, around 11.00, 27.40, 18.34, and 8.31% out of the total study region were highly, moderately, marginally and not suitable, respectively. The areas identified as highly and moderately suitable sites can be utilized for rice and coconut production. The major strength of the study is that open-source datasets were used with limited field survey making the analysis cost-effective. The insights from this study can optimize yield and income under the rice and coconut cultivation, benefiting growers, extension specialists, managers, policy-makers. In future, studies may be planned by employing more factors having higher spatial resolution with advanced geospatial techniques like machine learning, deep learning and statistics-based objective weighting methods to further enhance the reliability of crop suitability maps.

## Supplementary Information

Supplementary Information.

## Data Availability

All the data used in this manuscript is freely available from the sources given within the manuscript except soil depth and drainage which the authors don’t have the permission to share. Further, the datasets used in the current study available from the corresponding author on reasonable request.
