# Supplementary material for: Crop suitability analysis for the coastal region of India through fusion of remote sensing, geospatial analysis and multi-criteria decision making
Source: Sci Rep. 2025 Mar 13;15:8727. doi: 10.1038/s41598-025-90754-1 (PMC11906854; doi:10.1038/s41598-025-90754-1)
Supplement: Supplementary file 1 — Supplementary Information. [file 41598_2025_90754_MOESM1_ESM.docx]

**Supplementary table 1:** Rice parameter’s subclasses pairwise comparison matrix.

| **Elevation** | **λ_max_ = 4.140 CI = 0.047 RI = 0.9 CR = 0.052** | | | | | | |  |
| --- | --- | --- | --- | --- | --- | --- | --- | --- |
|  | <100 | | 100-500 | 500-1000 | >1000 |  |  |  |
| <100 | 1 | | 3 | 5 | 9 |  |  |  |
| 100-500 | 1/3 | | 1 | 3 | 5 |  |  |  |
| 500-1000 | 1/5 | | 1/3 | 1 | 3 |  |  |  |
| >1000 | 1/9 | | 1/5 | 1/3 | 1 |  |  |  |
| **Slope (in %)** | **λ_max_ = 5.326 CI = 0.081 RI = 1.12 CR = 0.073** | | | | | | |  |
|  | < 1 | | 1-3 | 3-10 | 10-15 | >15 |  |  |
| <1 | 1 | | 3 | 5 | 7 | 9 |  |  |
| 1-3 | 1/3 | | 1 | 2 | 5 | 7 |  |  |
| 3-10 | 1/5 | | 1/2 | 1 | 3 | 5 |  |  |
| 10-15 | 1/7 | | 1/5 | 1/3 | 1 | 2 |  |  |
| >15 | 1/9 | | 1/7 | 1/5 | 1/2 | 1 |  |  |
| **Depth (in cm)** | **λ_max_ = 7.409 CI = 0.068 RI = 1.32 CR = 0.052** | | | | | | |  |
|  | Very Deep | Deep | | Moderately deep | Moderately shallow | Shallow | Very shallow | Extremely shallow |
| Very Deep | 1 | 1 | | 2 | 3 | 5 | 7 | 9 |
| Deep | 1 | 1 | | 2 | 3 | 5 | 7 | 9 |
| Moderately deep | 1/2 | 1/2 | | 1 | 3 | 4 | 7 | 9 |
| Moderately shallow | 1/3 | 1/3 | | 1/3 | 1 | 2 | 5 | 7 |
| Shallow | 1/5 | 1/5 | | 1/4 | 1/2 | 1 | 2 | 4 |
| Very shallow | 1/7 | 1/7 | | 1/7 | 1/5 | 1/2 | 1 | 2 |
| Extremely shallow | 1/9 | 1/9 | | 1/9 | 1/7 | 1/4 | 1/2 | 1 |
| **Texture** | **λ_max_ = 5.108 CI = 0.027 RI = 1.12 CR = 0.024** | | | | | | |  |
|  | Clayey | | Loam | Sandy loam | Loamy sand | Sand |  |  |
| Clayey | 1 | | 3 | 5 | 5 | 9 |  |  |
| Loam | 1/3 | | 1 | 2 | 2 | 7 |  |  |
| Sandy loam | 1/5 | | 1/2 | 1 | 1 | 3 |  |  |
| Loamy sand | 1/5 | | 1/2 | 1 | 1 | 2 |  |  |
| Sand | 1/9 | | 1/7 | 1/3 | 1/2 | 1 |  |  |
| **Drainage** | **λ_max_ = 6.293 CI = 0.059 RI = 1.24 CR = 0.047** | | | | | | |  |
|  | Imperfect | | Very poor | Poor | Moderately well drained | Well drained | Excessive | |
| Imperfect | 1 | | 2 | 3 | 5 | 7 | 9 |  |
| Very poor | 1/2 | | 1 | 2 | 4 | 7 | 9 |  |
| Poor | 1/3 | | 1/2 | 1 | 3 | 5 | 7 |  |
| Moderately well drained | 1/5 | | 1/4 | 1/3 | 1 | 2 | 4 |  |
| Well drained | 1/7 | | 1/7 | 1/5 | 1/2 | 1 | 2 |  |
| Excessive | 1/9 | | 1/9 | 1/7 | 1/4 | 1/2 | 1 |  |
| **SOC (in %)** | **λ_max_ = 5.133 CI = 0.033 RI = 1.12 CR = 0.030** | | | | | | |  |
|  | > 1.00 | | 0.75-1.00 | 0.50-0.75 | 0.25-0.50 | <0.25 |  |  |
| >1.00 | 1 | | 2 | 3 | 5 | 9 |  |  |
| 0.75-1.00 | 1/2 | | 1 | 3 | 5 | 7 |  |  |
| 0.50-0.75 | 1/3 | | 1/3 | 1 | 2 | 4 |  |  |
| 0.25-0.50 | 1/5 | | 1/5 | 1/2 | 1 | 2 |  |  |
| < 0.25 | 1/9 | | 1/7 | 1/4 | 1/2 | 1 |  |  |
| **pH** | **λ_max_ = 5.222 CI = 0.055 RI = 1.12 CR = 0.049** | | | | | | |  |
|  | 7.5-8.5 | | 6.5-7.5 | 5.5-6.5 | 4.5-5.5 | <4.5 |  |  |
| 7.5-8.5 | 1 | | 3 | 3 | 5 | 3 |  |  |
| 6.5-7.5 | 1/3 | | 1 | 3 | 1 | 5 |  |  |
| 5.5-6.5 | 1/3 | | 1/3 | 1 | 3 | 7 |  |  |
| 4.5-5.5 | 1/5 | | 1 | 1/3 | 1 | 5 |  |  |
| <4.5 | 1/3 | | 1/5 | 1/7 | 1/5 | 1 |  |  |
| **Rainfall** | **λ_max_ = 4.009 CI = 0.003 RI = 0.9 CR = 0.003** | | | | | | |  |
|  | >1100 | | 900-1100 | 750-900 | < 750 |  |  |  |
| >1100 | 1 | | 2 | 5 | 9 |  |  |  |
| 900-1100 | 1/2 | | 1 | 3 | 5 |  |  |  |
| 750-900 | 1/5 | | 1/3 | 1 | 2 |  |  |  |
| <750 | 1/9 | | 1/5 | 1/2 | 1 |  |  |  |
| **Temperature** | **λ_max_ = 5.239 CI = 0.060 RI = 1.12 CR = 0.053** | | | | | | |  |
|  | 30-35 | | 25-30 | 20-25 | 15-20 | <15 |  |  |
| >30 | 1 | | 2 | 5 | 7 | 9 |  |  |
| 25-30 | 1/2 | | 1 | 2 | 5 | 8 |  |  |
| 20-25 | 1/5 | | 1/2 | 1 | 3 | 5 |  |  |
| 15-20 | 1/7 | | 1/5 | 1/3 | 1 | 2 |  |  |
| <15 | 1/9 | | 1/8 | 1/5 | 1/2 | 1 |  |  |

**Supplementary table 2:** Coconut parameter’s subclasses pairwise comparison matrix.

| **Elevation (m)** | **λ_max_ = 4.140 CI = 0.047 RI = 0.9 CR = 0.052** | | | | | |
| --- | --- | --- | --- | --- | --- | --- |
|  | <100 | 100-600 | 600-900 | >900 |  |  |
| <100 | 1 | 3 | 5 | 9 |  |  |
| 100-600 | 1/3 | 1 | 3 | 5 |  |  |
| 600-900 | 1/5 | 1/3 | 1 | 3 |  |  |
| >900 | 1/9 | 1/5 | 1/3 | 1 |  |  |
| **Slope (%)** | **λ_max_ = 5.326 CI = 0.081 RI = 1.12 CR = 0.073** | | | | | |
|  | <4 | 4-8 | 8-15 | 15-30 | >30 |  |
| <4 | 1 | 3 | 5 | 7 | 9 |  |
| 4-8 | 1/3 | 1 | 2 | 5 | 7 |  |
| 8-15 | 1/5 | 1/2 | 1 | 3 | 5 |  |
| 15-30 | 1/7 | 1/5 | 1/3 | 1 | 2 |  |
| >30 | 1/9 | 1/7 | 1/5 | 1/2 | 1 |  |
| **Depth (cm)** | **λ_max_ = 4.118 CI = 0.039 RI = 0.9 CR = 0.044** | | | | | |
|  | >100 | 75-100 | 50-75 | <50 |  |  |
| >100 | 1 | 2 | 5 | 7 |  |  |
| 75-100 | 1/2 | 1 | 3 | 5 |  |  |
| 50-75 | 1/5 | 1/3 | 1 | 3 |  |  |
| <50 | 1/7 | 1/5 | 1/3 | 1 |  |  |
| **Texture** | **λ_max_ = 3.014 CI = 0.007 RI = 0.58 CR = 0.012** | | | | | |
|  | Loam | SL, SiC, Si | S, C, LS |  |  |  |
| Loam | 1 | 3 | 7 |  |  |  |
| SL, SiC, Si | 1/3 | 1 | 3 |  |  |  |
| S, C, LS | 1/7 | 1/3 | 1 |  |  |  |
| **Drainage** | **λ_max_ = 5.371 CI = 0.093 RI = 1.12 CR = 0.083** | | | | | |
|  | Well drained | Moderately well drained | Imperfect | Excessive | Poorly drained |  |
| Well drained | 1 | 3 | 5 | 5 | 7 |  |
| Moderately well drained | 1/3 | 1 | 3 | 3 | 5 |  |
| Imperfect | 1/5 | 1/3 | 1 | 1 | 5 |  |
| Excessive | 1/55 | 1/3 | 1 | 1 | 3 |  |
| Poorly drained | 1/7 | 1/5 | 1/3 | 1/3 | 1 |  |
| **SOC (%)** | **λ_max_ = 5.133 CI = 0.033 RI = 1.12 CR = 0.030** | | | | | |
|  | >1.00 | 0.75-1.00 | 0.50-0.75 | 0.25-0.50 | <0.25 |  |
| >1.00 | 1 | 2 | 3 | 5 | 9 |  |
| 0.75-1.00 | 1/2 | 1 | 3 | 5 | 7 |  |
| 0.50-0.75 | 1/3 | 1/3 | 1 | 2 | 4 |  |
| 0.25-0.50 | 1/5 | 1/5 | 1/2 | 1 | 2 |  |
| <0.25 | 1/9 | 1/7 | 1/4 | 1/2 | 1 |  |
| **pH** | **λ_max_ = 4.191 CI = 0.064 RI = 0.9 CR = 0.071** | | | | | |
|  | 5.1-6.5 | 6.6-7.5, 4.5-5.0 | | 7.6-8.5, 4.0-4.4 | < 4.0 | |
| 5.0-6.5 | 1 | 3 | | 5 | 9 | |
| 6.5-7.5, 4.5-5.0 | 1/3 | 1 | | 3 | 7 | |
| 7.5-8.5, 4.0-4.5 | 1/5 | 1/3 | | 1 | 3 | |
| <4.0 | 1/9 | 1/7 | | 1/3 | 1 | |
| **Rainfall** | **λ_max_ = 4.009 CI = 0.003 RI = 0.9 CR = 0.003** | | | | | |
|  | >1500 | 1000-1500 | 500-1000 | <500 |  |  |
| >1500 | 1 | 2 | 5 | 9 |  |  |
| 1000-1500 | 1/2 | 1 | 3 | 5 |  |  |
| 500-1000 | 1/5 | 1/3 | 1 | 2 |  |  |
| <500 | 1/9 | 1/5 | 1/2 | 1 |  |  |
| **Temperature** | **λ_max_ = 2.000 CI = 0.00 RI = 0.00 CR = 0.00** | | | | | |
|  | 26-29 | <26 |  |  |  |  |
| 26-29 | 1 | 3 |  |  |  |  |
| <26 | 1/3 | 1 |  |  |  |  |

**Supplementary table 3:** Area under LULC map after reclassification.

| **S. No.** | **Class** | **Area (ha)** | **Area (%)** |
| --- | --- | --- | --- |
| 1 | Cropland area | 21390891.48 | 65.05 |
| 2 | Non-cropland area | 11492478.98 | 34.95 |
|  | Total | 32883370.46 | 100 |
